# cazy_webscraper: local compilation and interrogation of comprehensive CAZyme datasets

**DOI:** 10.1099/mgen.0.001086

**Published:** 2023-08-14

**Authors:** Emma E. M. Hobbs, Tracey M. Gloster, Leighton Pritchard

**Affiliations:** ^1^​ School of Biology and Biomedical Sciences Research Complex, University of St Andrews, North Haugh, St Andrews, Fife, KY16 9ST, UK; ^2^​ Strathclyde Institute of Pharmacy and Biomedical Sciences, University of Strathclyde, Glasgow, G4 0RE, UK; ^3^​ Cell and Molecular Sciences, James Hutton Institute, Invergowrie, Dundee, DD2 5DA, UK

**Keywords:** CAZy, CAZymes, database, lignocellulose, software, carbohydrate active enzymes

## Abstract

Carbohydrate active enzymes (CAZymes) are pivotal in biological processes including energy metabolism, cell structure maintenance, signalling, and pathogen recognition. Bioinformatic prediction and mining of CAZymes improves our understanding of these activities and enables discovery of candidates of interest for industrial biotechnology, particularly the processing of organic waste for biofuel production. CAZy (www.cazy.org) is a high-quality, manually curated, and authoritative database of CAZymes that is often the starting point for these analyses. Automated querying and integration of CAZy data with other public datasets would constitute a powerful resource for mining and exploring CAZyme diversity. However, CAZy does not itself provide methods to automate queries, or integrate annotation data from other sources (except by following hyperlinks) to support further analysis. To overcome these limitations we developed cazy_webscraper, a command-line tool that retrieves data from CAZy and other online resources to build a local, shareable and reproducible database that augments and extends the authoritative CAZy database. cazy_webscraper’s integration of curated CAZyme annotations with their corresponding protein sequences, up-to-date taxonomy assignments, and protein structure data facilitates automated large-scale and targeted bioinformatic CAZyme family analysis and candidate screening. This tool has found widespread uptake in the community, with over 35 000 downloads (from April 2021 to June 2023). We demonstrate the use and application of cazy_webscraper to: (i) augment, update and correct CAZy database accessions; (ii) explore the taxonomic distribution of CAZymes recorded in CAZy, identifying under-represented taxa and unusual CAZy class distributions; and (iii) investigate three CAZymes having potential biotechnological application for degradation of biomass, but lacking a representative structure in the PDB database. We describe in general how cazy_webscraper facilitates functional, structural and evolutionary studies to aid identification of candidate enzymes for further characterization, and specifically note that CAZy provides supporting evidence for recent expansion of the Auxiliary Activities (AA) CAZy family in eukaryotes, consistent with functions potentially specific to eukaryotic lifestyles.

## Data Summary

The authors confirm all supporting data, code and protocols have been provided within the article or through supplementary data files.

**Cazy_webscraper software:**

Project name: cazy_webscraper

Project home page: https://hobnobmancer.github.io/cazy_webscraper/

GitHub Repository: https://github.com/HobnobMancer/cazy_webscraper.

Documentation: https://cazy-webscraper.readthedocs.io/.

DOI: https://doi.org/10.5281/zenodo.6343936

Operating systems: Linux, MacOS and Windows 7 or higher.

Installation: GitHub (from source), Bioconda, Pypi

Programming language: Python 3.9.

Licence: MIT Licence.

Any restrictions to use by non-academics: None.

**Supplementary/additional data files:**

All additional files are available in a supplementary GitHub repository. A list (including file name, file type, size and description) is provided in the GitHub repository.

Download link (to download zip file containing all additional files): https://github.com/HobnobMancer/SI_Hobbs_et_al_2023_cazywebscraper/raw/master/additional_files.zip

Project home page: https://hobnobmancer.github.io/SI_Hobbs_et_al_2023_cazywebscraper/

GitHub Repository: https://github.com/HobnobMancer/SI_Hobbs_et_al_2023_cazywebscraper

DOI: https://doi.org/10.5281/zenodo.7768336

**Impact Statement**

Carbohydrate active enzymes (CAZymes) are pivotal in many biological processes including cell signalling, infection, and the structural integrity of organisms such as plants and bacteria, and are mined bioinformatically for industrial biotechnology. The CAZy database is the most comprehensive resource for CAZyme data and classification, but does not provide methods to automate queries, or integrate data from other sources to aid identification of CAZymes of biological or industrial interest. To overcome these limitations we developed cazy_webscraper, a command-line tool that retrieves data from CAZy and other online resources to build a local, shareable and reproducible database that augments and extends the authoritative CAZy database. This tool has found widespread uptake in the community, with over 35 000 downloads from April 2021 to June 2023. We demonstrate how cazy_webscraper facilitates functional, structural and evolutionary studies of large CAZyme datasets to identify candidate enzymes for further characterization. It provides a powerful tool to researchers, complementing other packages in the carbohydrate enzyme field, to enable bioinformatic analysis of CAZymes in the ever-expanding world of genomic data.

## Introduction

Carbohydrate active enzymes (CAZymes) catalyse modification, synthesis and degradation of polysaccharides and glycoconjugates [1]. They are vital in many biological pathways including metabolism and cell signalling, are used for industrial bioprocessing of organic materials, and are of fundamental research interest [2–5].

The most comprehensive and authoritative CAZyme resource is the manually curated CAZy database (www.cazy.org). CAZy is built by classification of sequenced CAZymes (glycoside hydrolases, GHs; glycosyltransferases, GTs; polysaccharide lyases, PLs; carbohydrate esterases, CEs; auxiliary activities, AAs; and non-catalytic carbohydrate binding modules, CBMs) into sequence similarity-based families corresponding to presumed shared mechanism and structural fold, enabling principled annotation and prediction of enzyme function [6]. This database is widely used as the primary reference database for bioinformatic analyses of CAZymes. For many downstream analyses and applications using CAZy data, including genome annotation and the training of machine learning models, it is necessary to be able to filter sequences, often incorporating additional data held in other resources. However, CAZy currently provides most of its data only in two forms: a plain text download containing all members for each CAZy family; and via a browser interface, paginated in small groups for a subset of functionally and/or structurally characterized members of the family. The absence of a public application programming interface (API) or query function makes automated retrieval and querying of large and/or user-filtered datasets from CAZy for bioinformatic analysis challenging for scientists who cannot program.

Several software tools have been developed to overcome the limitations of the CAZy web interface, but many are no longer supported or have not been updated to reflect recent changes to the CAZy site structure [6]. One such deprecated tool is CAZy_utils (https://github.com/nielshanson/CAZy_utils) which was similar in function to cazy_webscraper in that it also built a comprehensive local CAZyme database from the available CAZy data. Other tools retrieve only limited data from CAZy: cazyseqs (https://github.com/walshaw/cazyseqs) gathers protein sequences from CAZy families, but not CAZy family annotations; and cazy_parser [7] compiles HTML tables from the CAZy website into a single comma-separated variable (CSV) file.

To fill this gap in capability, and to facilitate downstream analyses using CAZy data, we present cazy_webscraper (https://doi.org/10.5281/zenodo.6343936), a command-line tool that automates data retrieval from the CAZy database and other resources, integrating these into a local SQLite3 database. cazy_webscraper has received significant community uptake, averaging 1900 downloads per month (January–June 2023 [8]), though we note that package download quotas do not always directly correlate with usage as automated retrieval may artificially inflate the count. However, community activity in the repository and issue tracker also indicates uptake by the wider community. cazy_webscraper can integrate protein sequence, taxonomic lineage, Enzyme Commission (EC) annotation and structural information data from public repositories including the National Center for Biotechnology Information (NCBI) [9], UniProt [10], Research Collaboratory for Structural Protein Data Bank (RSCB PDB) [11] and the Genome Taxonomy Database (GTDB) [12]. All data retrieval is logged for audit and reproducibility. The database constructed by cazy_webscraper is reusable and shareable, enabling replication of downstream bioinformatic analyses. cazy_webscraper also provides an API to this database, enabling integration into automated analyses and other software tools.

We demonstrate the use and application of cazy_webscraper with practical examples. We survey taxonomic diversity in CAZy, highlighting uneven representation of archaeal lineages and CAZy families to identify scope for strategic investigations to extend our understanding of these enzymes. We analyse sequence diversity across the PL20 family, identifying a conserved enzyme represented only in *Streptomyces *, and a β-1,4-glucuronan lyase (EC 4.2.2.14) from *Galbibacter* sp. BG1 that may be the only membrane-associated PL20 glucuronan lyase currently represented in CAZy. In addition, we survey the known structural representation of CEs in CAZy, and identify and investigate two CAZyme groups that currently have no structural representatives in the RCSB PDB and which are of potential interest to industrial biotechnology.

## Methods

### Implementation of cazy_webscraper

**Fig. 1.**
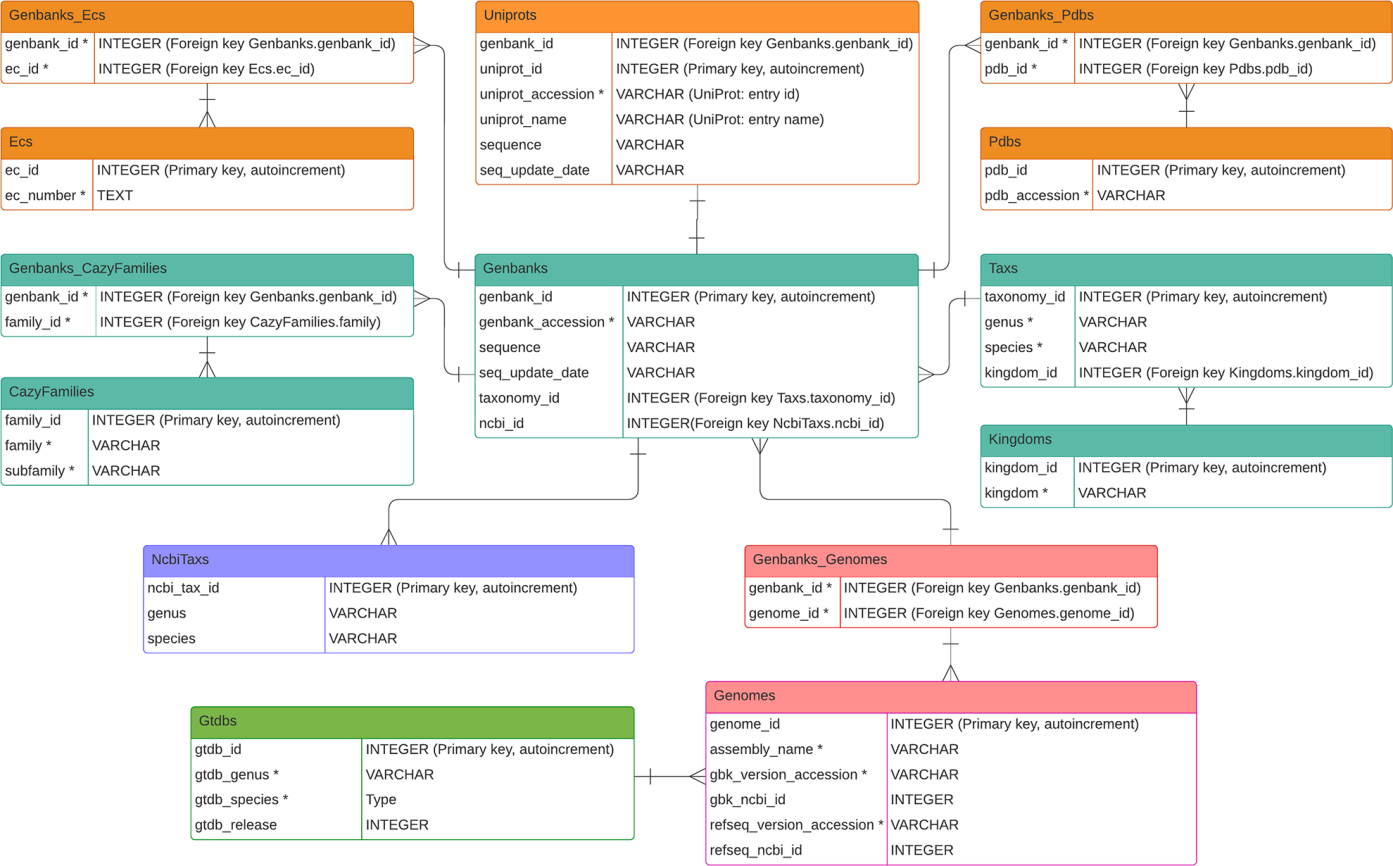
Entity-relationship (ER) model of the cazy_webscraper database structure. Teal boxes represent tables containing data collected from CAZy. Orange tables contain data retrieved from UniProtKB. Purple and pink boxes represent tables containing data retrieved from NCBI, and the green table contains data retrieved from GTDB. The datatype VARCHAR represents a variable-length character string. Fields under UNIQUE constraint are marked with an asterisk, and such fields within the same table are under joint UNIQUE constraint.

cazy_webscraper is implemented as a Python package. Its core function is to automate retrieval of records from the public CAZy database, and construct a corresponding local SQLite3 database [13] (schema shown in Fig. 1) suitable for integration of additional sequence, structure and annotation data. Object relational mapping (ORM) is specified and managed using SQLAlchemy [14].

CAZy provides a plain text file dump of the complete database for public use. The CAZy download can be parsed by cazy_webscraper to extract all CAZyme records, or only records matching user-specified criteria. The CAZy dump downloaded by cazy_webscraper is cached locally to enable multiple distinct operations (such as filtering) to be applied reproducibly on the original dataset. This facilitates reproducible generation of specialized databases, such as separate databases for each CAZy class, or for a specified range of organisms. cazy_webscraper imports CAZy fields including GenBank accessions, source organism taxon (genus, species and kingdom) and CAZy family. At the time of writing (June 2023), PubMed IDs and clan data (an additional CAZy classification encompassing groups of families that share a fold and catalytic machinery) [6] are not provided in the downloadable database dump, so are not imported.

cazy_webscraper identifies distinct CAZymes uniquely by their NCBI accession as recorded in CAZy. These are mostly GenBank accessions, although some entries in CAZy are recorded as RefSeq accessions. A single CAZyme may be associated with more than one CAZy family or subfamily classification, and the cazy_webscraper database eliminates some redundancy present in the CAZy dataset on import.

When run, cazy_webscraper will, by default, construct a new database but can also update an existing local database. Updating a local database using different filters on the same CAZy database dump, or a series of successive CAZy database downloads, does not introduce duplicate CAZyme records. cazy_webscraper caches unprocessed data as they are retrieved from CAZy, or from external databases such as UniProt, NCBI and GTDB. If a download is interrupted, cazy_webscraper can be resumed from the point it halted. Operations involving retrieval of data from NCBI (including taxonomic, genomic and sequence data) use the Entrez interface and therefore require the user to provide an email address [15].

### Filtering CAZy data on import

The publicly available CAZy database download contains the complete set of CAZyme records, but not all data available at the CAZy web service. cazy_webscraper filtering options can be used to import into the local database only records matching combinations of user-specified criteria, including: CAZy class, CAZy family, CAZy subfamily, kingdom, genus, species and/or strain. Filters can be specified using a configuration file, or by providing parameters at the command-line. Similar filters, and an additional EC number filter, can be used to control the retrieval of data from external databases such as UniProt, NCBI, PDB and GTDB. Table 1 lists example commands and a summary of the data imported.

**Table 1.** Example commands that import data from the CAZy, UniProt, NCBI GenBank and RCSB PDB databases, or perform data export

Example command	Result
cazy_webscraper my_email@domain.com	Download whole CAZy database and import all data into the local database
cazy_webscraper my_email@domain.com --families PL20,PL28 --kingdoms bacteria	Download whole CAZy database but import only CAZymes from CAZy families PL20 and PL28 that are derived from bacteria into the local database
cw_get_ncbi_taxs cazyme_db.db my_email@domain.com --families GH3 --kingdoms archaea	Download and import into the local database (cazyme_db.db) all taxonomy lineages from NCBI for CAZymes in family GH3 that are derived from archaea
cw_get_genomic_data cazyme_db.db my_email@domain.com --classes CE,AA --kingdoms viruses	Download and import into the local database (cazyme_db.db) all genomic IDs and version accessions for CE and AA CAZymes that are derived from viruses
cw_get_gtdb_taxs cazyme_db.db --kingdoms bacteria	Download and import into the local database (cazyme_db.db) all taxonomy lineages from GTDB for CAZymes derived from bacteria
cw_get_uniprot_data cazyme_db.db --classes PL --ec --pdb --sequence	Download and import into the local database (cazyme_db.db) all UniProt IDs, protein names, EC numbers, PDB IDs and protein sequences from UniProt for all PL CAZymes
cw_get_genbank_seqs my_email@domain.com cazyme_db.db --classes GH --kingdoms bacteria	Download and import into the local database (cazyme_db.db) all protein sequences from NCBI for all GH CAZymes derived from bacterial species
cw_get_pdb_structures cazyme_db.db --classes GT --species ‘Trichoderma reesei’ --strains ‘Aspergillus flavus AF13’	Download all RCSB PDB IDs in the local database corresponding to GT CAZymes derived from all strains of *Trichoderma reesei* and from the *Aspergillus flavus* strain AF13
cw_extract_db_seqs cazyme_db.db genbank --kingdoms archaea --fasta_file archaea_seqs.fasta	Write a multisequence FASTA file comprising GenBank protein sequences that are derived from archaea and that are stored in the local database cazyme_db.db
cw_query_database cazyme_db.db --classes GH,CBM --ec --pdb	Write a .csv file comprising (i) the GenBank accessions of all CAZymes, (ii) EC number annotations, and (iii) PDB IDs of CAZymes in the GH and CBM classes that are stored in the local database cazyme_db.db

### Automated retrieval of additional protein data, genomic data, sequences and structures

cazy_webscraper can extend the local CAZy database to incorporate protein sequences, functional annotations and structural data. cazy_webscraper uses NCBI accessions to retrieve corresponding genome IDs, protein sequences and other data for each CAZyme using Biopython [16] and Entrez [15]. UniProt data (UniProt ID, protein name, protein sequence, PDB ID, EC number, etc.) are retrieved using the BioServices package [17].

cazy_webscraper can update local CAZyme protein sequences if it detects a more recent version available from UniProt or NCBI. Protein sequences can be exported from the local database to FASTA format, or to a BLAST+ database (using the --blastdb flag).

PDB IDs obtained from UniProt can be used to obtain protein structures from RCSB PDB using Biopython [18]. PDB structure files are not stored directly in the database but are saved to a user-specified location in the user’s preferred format (mmCIF, pdb, xml, mmtf or bundle). Database storage of structural data (as blobs and/or plain text) is planned in the cazy_webscraper development roadmap.

**Automated retrieval of NCBI and GTDB taxonomic classifications**

cazy_webscraper uses the NCBI accession for each CAZyme record to retrieve and import current taxonomic assignment directly from the NCBI Taxonomy [19] and/or GTDB databases [12]. To link successfully with GTDB, an NCBI genome assembly must be present in the GTDB database and associated with the corresponding CAZyme accession.

**Querying the local CAZyme database**

cazy_webscraper provides a command-line interface for common queries, returning output in CSV and/or JSON format. By default, the program returns only the NCBI accession for each CAZyme matching the provided criteria. The --include option allows reporting of additional fields including: CAZy class; CAZy family; CAZy subfamily; taxonomic kingdom; genus; host organism; GenBank protein sequence; UniProt accession; protein name; EC number; PDB accessions; and UniProt protein sequence.

The database generated by cazy_webscraper can also be queried directly by advanced users, *via* the SQLite console, or by connecting to the database programmatically.

**Reproducible and shareable datasets and documentation**

cazy_webscraper logs all data retrievals in the local database. The single compact database generated is time-stamped and shareable, facilitating reproduction of the downstream analyses that use it.

Data retrieval can be configured using a YAML file for precise reproduction. Reproducible local reconstruction of the database is assisted by caching unprocessed data from each data source (CAZy, NCBI, etc.). Each release of cazy_webscraper is associated with a unique digital object identifier (DOI), assigned by Zenodo.

**Installation**

cazy_webscraper can be installed using Bioconda [20] or PyPI [21] package managers, or from source code (https://github.com/HobnobMancer/cazy_webscraper). cazy_webscraper is released under the MIT open licence.

**Data generation and analysis**

cazy_webscraper version 2.0.13 (DOI: 10.5281/zenodo.6343936) was used for all examples in this paper. All additional files are available in the GitHub repository: https://github.com/HobnobMancer/SI_Hobbs_et_al_2023_cazywebscraper (DOI: 10.5281/zenodo.7768336), which includes a README file walk-through and a description of all additional files.

All bash commands used to augment, query and extract data from the local CAZyme database are provided as executable bash scripts in additional file 20, alongside a README file walk-through. The figures in this paper were generated using an RMarkdown notebook (additional file 1 is an archive including this notebook and all data files required to run the analyses). All analyses were performed on a consumer-grade laptop with AMD FX-6300 (3.5 GHz) processor, 16 GB RAM, and an onboard SSD drive and theoretical network speed of 107 Mbps, running Ubuntu 22.04.2 LTS. All timings were measured in triplicate, and variation is reported as standard deviation. For all SQL commands executed in the SQLite3 console the number of unique protein sequence accessions returned by the query was interpreted as the count of CAZymes matching the query. Default parameters were used for all software, unless otherwise stated.

### Building the local reference CAZyme database

The CAZy database was downloaded from http://www.cazy.org on 13 January 2022 and used to create a local database with cazy_webscraper, configured using the buil_atabas.sh script (additional file 20).

### Taxonomic assignments in the local CAZyme database

During testing, we found that some CAZy database records were assigned to taxa having no corresponding entry in the NCBI Taxonomy database. Additional file 21 lists 108 proteins annotated as deriving from more than one source organism in the July 2022 CAZy release.

The number of CAZymes per taxonomic kingdom as recorded in CAZy, and the count of CAZymes per taxonomic kingdom for each CAZy class and each CAZy family, were obtained with the script cazymes_per_cazy_kingdom.sh (additional file 20). Additional NCBI taxonomic assignments were imported using the script get_ncbi_taxs.sh (additional file 20).

The count of proteins in the NCBI GenBank database per kingdom was retrieved from the NCBI Taxonomy database (https://www.ncbi.nlm.nih.gov/taxonomy accessed May 2022). A χ^2^ test (additional file 1) was used to test whether the distribution of CAZymes per taxonomic kingdom recorded in CAZy differed from that in the NCBI protein database. A matrix containing the explained variance per kingdom was calculated for this χ^2^ model (additional file 1) using the equation:

(1) $$100\left(\frac{Pearsonresiduals}{X^{2}teststatistic}\right)$$

Pearson residuals are defined using the equation below, where *O* represents observed values and *E* represents expected values:

(2) $$\frac{\left(O-E\right)}{\surd E}$$

A second χ^2^ test was used to test whether the distribution of CAZymes per taxonomic kingdom in CAZy families dominated by eukaryotes differed from that in CAZy families dominated by bacteria; a matrix containing the explained variance per kingdom was calculated for this χ^2^ model (additional file 1).

Lineage information and the count of CAZymes in each NCBI taxon were obtained for all archaeal CAZymes in the local database using the script get_archaeal_cazymes.sh (additional file 20). Incomplete lineages were manually removed from the data, as were all lineages assigned as *Candidatus*. Additional file 22 lists all archaeal NCBI lineages in the resulting local database. These remaining assignments were used with RAWGraphs version 2.0 [22], to construct alluvial diagrams of the number of CAZymes annotated in CAZy at each node of the archaeon lineage.

### Survey of sequence diversity in the PL20 family

Protein sequences of CAZymes in family PL20 were downloaded from NCBI and added to the local CAZyme database using cazy_webscraper (get_pl20_seqs.sh, additional file 20). The sequences were extracted to a multi-sequence FASTA file (additional file 23). All-vs-all pairwise sequence comparison was performed with these sequences using BLASTP+ v2.13.0 [23] (blastp_pl20.sh, additional file 20). Corresponding taxon assignments were extracted from the local database (get_pl20_taxons.sh, additional file 20). Using additional file 1, pairwise sequence similarity across the family was calculated as the BLAST Score Ratio (BSR) [24] for each top-ranked pairwise alignment, and the R package heatmaply v1.3.0 [25] was used to cluster the resulting matrix (euclidean distance, hclust function). Bar charts were produced plotting the taxonomic kingdom distribution across each axis of the heatmap using ggplot2 v3.3.5 [26].

The HX109_05010 (HX109, GenBank accession QLE00955.1) protein sequence was queried using BLASTP+ v2.13.0, with the BLOSUM45 matrix, against the NCBI non-redundant (nr) protein database (accessed 5 May 2022), to identify candidate conserved functional domains (additional file 8).

### Prediction of signal peptide and transmembrane domains

Signal peptides were predicted using SignalP (version 6.0 [27]). Transmembrane domains were predicted using DeepTMHMM, *via* the DeepTMHMM server (https://dtu.biolib.com/DeepTMHMM, version 1.0.15) [28]).

### Structural fold prediction and structure superimposition

Structural folds were predicted for individual domains of proteins (excluding signal peptides) using the official AlphaFold Colab notebook (version 2.1.0 [29]). Structures obtained from RCSB-PDB were superimposed onto the predicted structures and root mean square distance (RMSD) was calculated using the MatchMaker tool, followed by the Match-Align tool, in UCSF Chimera (version 1.16 [30, 31]).

### Structural survey of CE families

RSCB-PDB accessions for all CE class CAZymes in the local database were retrieved from UniProt (accessed April 2022). The number of CAZymes in each CE family with at least one PDB ID was obtained using the script get_ce_pdbs.sh (additional file 20).

Protein sequences for all CE family members were downloaded from NCBI, imported into the local database and written to a multi-sequence FASTA file [additional file 24 (CE19); additional file 25 (CE12)] using cazy_webscraper (get_ce_seqs.sh, additional file 20). These sequences were clustered using MMseqs2 (v13.45111 [32]) with thresholds of 40 % identity and 80 % coverage (cluster_ce12_ce19.sh, additional file 20). The resulting clusters containing proteins with corresponding structures deposited in RCSB-PDB were identified using a Python script (gather_clusters.py, additional file 20).

### Functional and structural prediction for CE19 enzyme TBR22_41900 (TBR22)

The full-length protein sequence of the CE19 CAZyme TBR22_41900 (TBR22, GenBank ID BCS34995.1) was chosen to represent a cluster of CE19 proteins identified by MMSeqs2 but having no structural representative in RCSB-PDB. The protein sequence of TBR22 was queried against all other CE19 family members in the local CAZyme database using BLASTP+ (blastp_ce19.sh, additional file 20; results in additional file 11). CAZy family domains were predicted for this sequence and for D6B99_08585 (AYD47656.1, additional file 12) using dbCAN v3.0.2 [33].

The C-terminal domain of TBR22 (residues 380–634) was queried against the NCBI nr protein database using the NCBI BLASTP+ server (results in additional file 13), and against the RSCB-PDB database, using the PDB server (both accessed June 2022).

An iterative sequence-based search for structural homologues was made using the HHpred server (v2.1, https://toolkit.tuebingen.mpg.de/tools/hhpred [34]) with the full-length TBR22 protein as query to the RCSB-PDB database (additional file 16). The structural fold of TBR22 was predicted using the AlphaFold Colab service (additional file 14). Structures obtained from the HHpred search were superimposed onto the predicted structure as described above (additional file 17).

Protein sequences for all CE enzymes with at least one representative PDB ID in the local CAZyme database were written to a multi-sequence FASTA file (get_ce19_pdb_prots.py, additional file 20). The C-terminal domain of TBR22 was queried against these sequences using BLASTP+ to identify potential homologues (blastp_cterm_ce19.sh, additional file 20; results in additional file 15).

### Annotation and structural fold prediction of CAZyme domains in HUW50_16055 (HUW50)

CAZyme domains were predicted for the protein HUW50_16055 (HUW50, QNF30995.1, additional file 26) using dbCAN (version 3.0.4). The full-length sequence of HUW50 was queried against the protein sequences of two structurally characterized CE12 CAZymes, three structurally characterized PL11 CAZymes and 16 structurally characterized CBM35 CAZymes using BLASTP+ (additional files 27–29; queries against the CBM35 sequences used the BLOSUM45 matrix). HUW50 CAZyme domains were annotated using dbCAN (HMMer results) and the BLASTP+ sequence alignments.

Structural folds were predicted separately for each predicted CAZyme domain in HUW50 using AlphaFold Colab (additional file 19), and experimentally derived structures were superimposed onto the corresponding HUW50 domain using UCSF Chimera as described above. Metal ion and substrate binding site annotations were manually retrieved from UniProt.

## Results

The complete CAZy database was downloaded and parsed, the data were compiled into a local SQLite3 database, and taxonomic lineages were recovered from the NCBI taxonomy database using cazy_webscraper in 14 min 53 s (±21 s). After excluding duplicate records, 2 232 090 unique CAZyme records were recovered. The resulting database was 270 MB in size and comprised proteins representing 199 076 taxa from five kingdom-level groups (bacteria, eukaryota, viruses, archaea and unclassified), spanning 688 CAZy families. A total of 108 records in the original CAZy database were found to be annotated with multiple inconsistent taxonomic lineages.

### Taxonomic distribution of CAZymes in CAZy

**Fig. 2.**
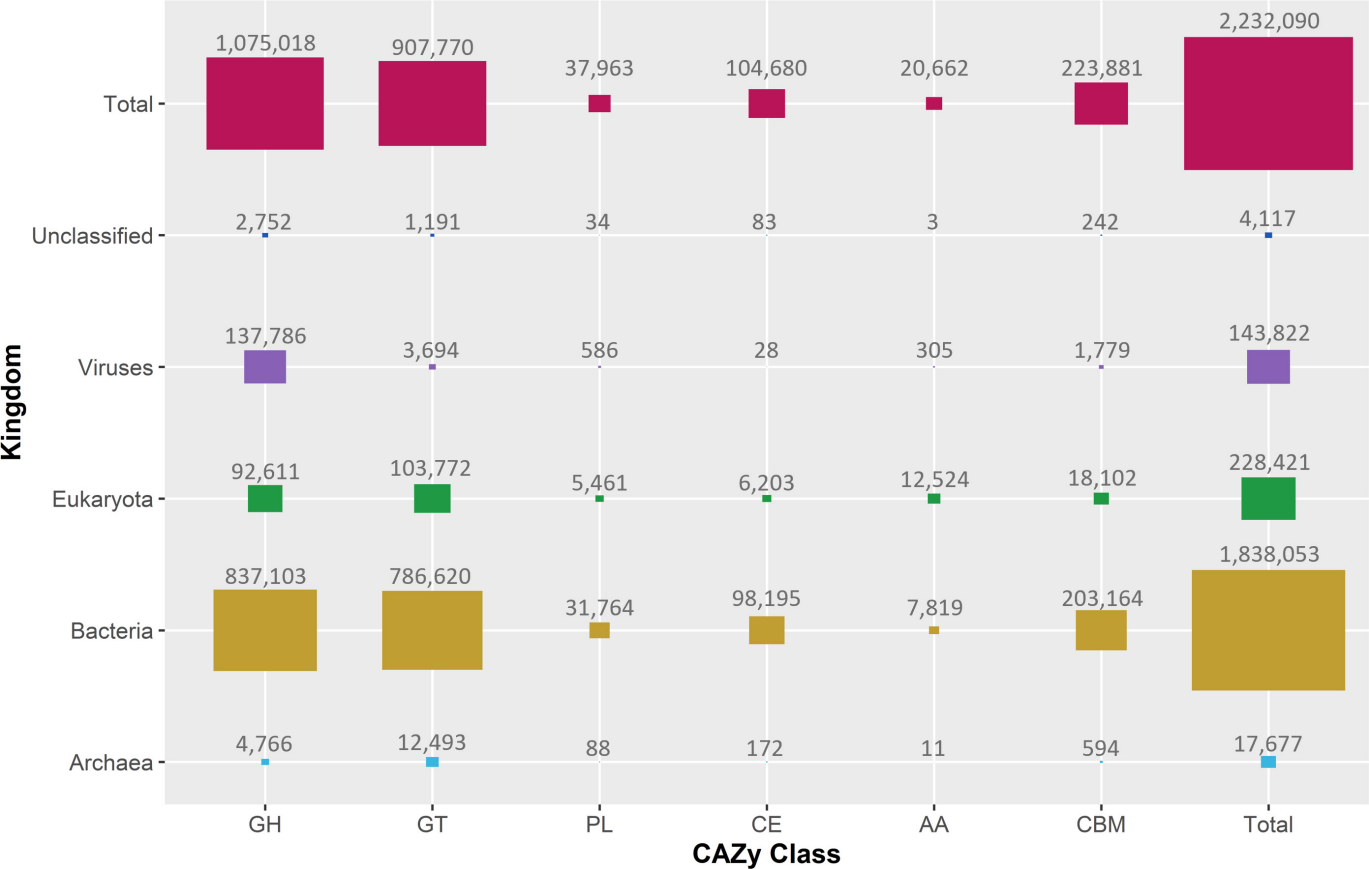
Proportional areas plot of the count of CAZymes in each CAZy class (total, and by taxonomic kingdom), as recorded in CAZy (January 2022). Numbers show the count of unique sequence IDs associated with each combination of kingdom and CAZy class. Note that a single CAZyme may be assigned to several classes.

The CAZy database server does not provide summary information describing the total number of unique CAZymes, or the total number of CAZymes, in each taxonomic group (e.g. domain or kingdom). We retrieved the total number of CAZymes per taxonomic kingdom (archaea, bacteria, eukaryotes, viruses and unclassified) for each CAZy class as described in the Methods (Fig. 2, time taken: 2 min 1 s, ±3 s).

### CAZyme distribution by kingdom differs between CAZy and NCBI

As all CAZy records are drawn from NCBI, we expected that NCBI’s underlying sampling bias by taxonomic kingdom, such as an overrepresentation of bacteria, might also be evident in CAZy. However, we found a statistically significant difference between the distributions of sequences across each kingdom in CAZy and in NCBI (χ^2^ test, *P*<2.2E-16). We conclude that kingdom-level bias in CAZy does not simply reflect an underlying sampling bias in NCBI, but may represent a kingdom-level difference in relative abundance of CAZymes, or variation in the accuracy or comprehensiveness of CAZyme annotation between kingdoms. We find specifically that ‘unclassified’, eukaryotic and archaeal sequences are relatively under-represented in CAZy, compared to what would be expected from the corresponding protein abundance in NCBI, but bacteria and viruses are over-represented (additional file 1).

### Relative and absolute representation of CAZymes varies by kingdom in CAZy

We wished to investigate how the relative abundance of CAZymes in each species varies between kingdoms in CAZy. We expect any variation potentially to derive from a combination of underlying sampling bias across kingdoms, differences in annotation quality and/or the differing prevalence of CAZymes in each kingdom. Table 2 presents kingdom-wise counts of: all species-level taxa in NCBI; the number of those taxa with at least one annotated CAZyme in CAZy; and the number of those taxa with at least 50 annotated CAZymes in CAZy. We find that less than 1.5 % (29 283) of all species in the NCBI Taxonomy database have any CAZymes represented in CAZy, and less than 0.5 % (6,693) of all species are represented by more than 50 records in CAZy. Eukaryotic species are especially under-represented in the CAZy database.

**Table 2.** Counts of species-level taxa in NCBI with at least one, and with at least 50, CAZymes represented in CAZy. The percentage of all species in the corresponding kingdom that this represents is also shown.

Kingdom	Species	At least 1 CAZyme		At least 50 CAZymes	
		Number	Per cent	Number	Per cent
Archaea	12 709	665	5.23	109	0.86
Bacteria	471 432	14 248	3.02	6128	1.30
Eukaryota	1 420 577	13698	0.96	456	0.03
Viruses	49 675	672	1.35	0	0
Total	1 954 393	29283	1.50	6693	0.34

We find that the abundance of CAZy records within a species also varies by kingdom. Forty per cent of bacterial species that have at least one CAZy record have more than 50 CAZy records, whereas this proportion falls to 16 % for archaea and 3 % for eukaryotes. No virus species is associated with more than 50 CAZy records, probably because of their restricted genome size. We interpret these data potentially to be indicative of differential expansion of CAZyme families within kingdoms, but with the caveat that there may be annotation quality differences between kingdoms.

### AA families are expanded in eukaryotes and dominated by eukaryotic sequences

**Fig. 3.**
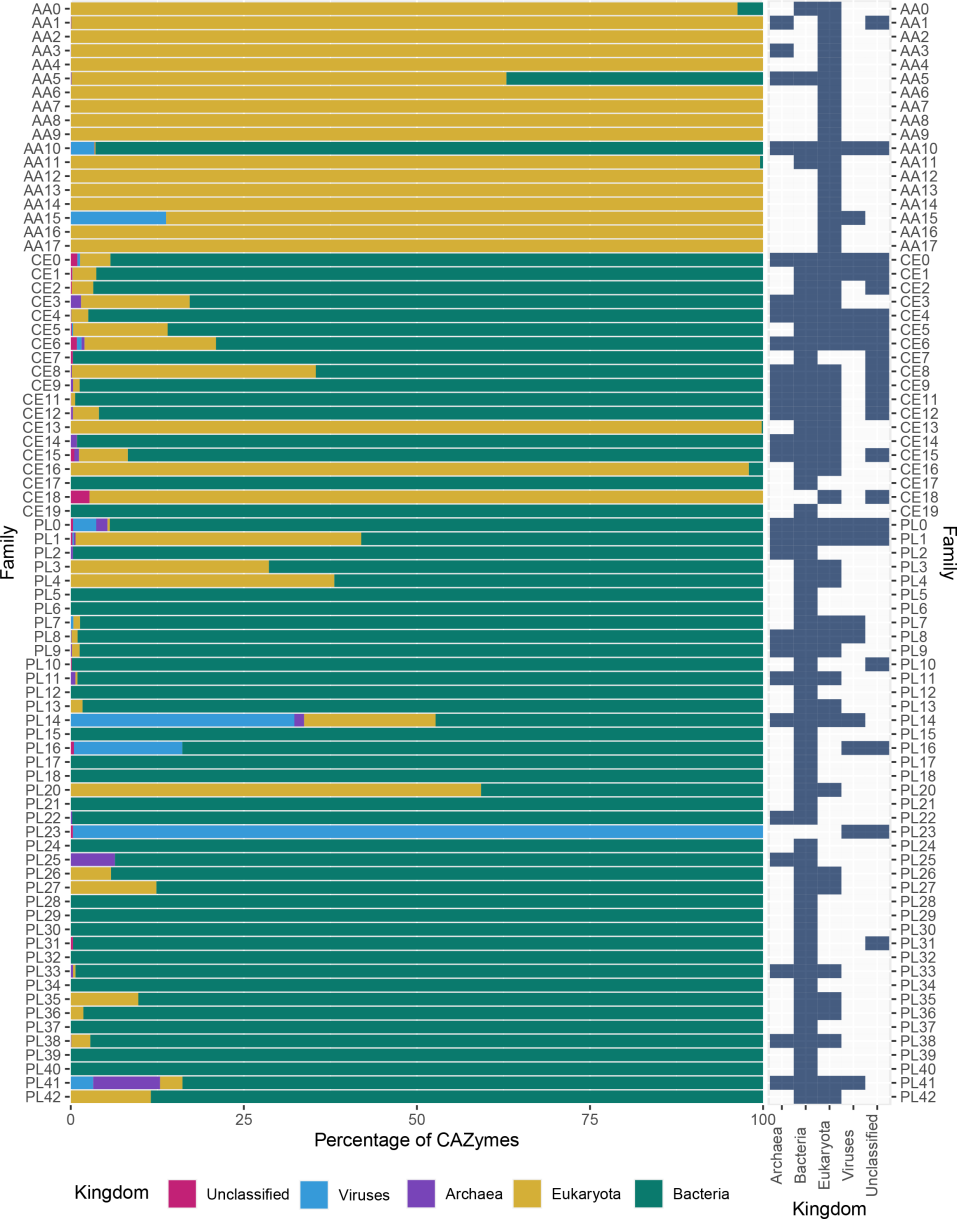
The proportion of CAZymes in each taxonomic kingdom for AA, CE and PL families (left), and absolute presence (blue)/absence (grey) in each kingdom (right). Families CE10 and PL19 are excluded as they have been withdrawn from the CAZy database.

Most records in CAZy (82.4 %) are bacterial CAZymes (Fig. 2). If CAZy classes were distributed evenly across all kingdoms, we would expect bacterial proteins to dominate in all cases. However, AA CAZymes in the CAZy database, including ligninolytic and lytic polysaccharide mono-oxygenases [6], are predominantly derived from eukaryotes. Seventeen of the 20 CE families, and 41 of the 43 PL families, are dominated by bacterial CAZymes. By contrast, 17 of the 18 AA families are dominated by eukaryotic enzymes, with several families only being represented at all in eukaryotes. Given the general under-representation of eukaryotic CAZymes in CAZy, we interpret this to reflect eukaryote-specific expansion of AA families, perhaps uniquely among CAZy classes. Kingdom-level distributions of all AA, CE and PL families are plotted in Fig. 3, and kingdom-level distributions for the complete set of CAZy families are plotted in additional file 2. Extending the comparison to all CAZy families (additional file 2; Supplementary Material 1Supplementary Material 1, Fig. S1), we find that 72 % (329) of all families are dominated by bacteria, and 23 % (105) of families contain a majority of eukaryotic sequences. We note that, while CAZy families dominated by bacterial sequences tend also to contain sequences from all other kingdoms (eukaryotes, archaea, viruses, unclassified), those families dominated by eukaryotic sequences have a different distribution across kingdoms (χ^2^ test, *P*=4.517E-15) and a tendency to contain only eukaryotic sequences. We believe that this may be consistent with a relatively recent expansion of CAZyme activities in eukaryotes, possibly in functions that are specifically beneficial for eukaryotic lifestyles, such as synthesis and degradation of chitin, lignin and cellulose.

### Identifying under-represented archaeal groups in CAZy

CAZy catalogues taxonomic kingdom, genus and species (and sometimes the strain) of each source organism, and further taxonomic detail is provided for each CAZy family by means of a Krona plot on the CAZy webserver. cazy_webscraper can retrieve the complete and most recent lineage from either or both of the NCBI Taxonomy and GTDB databases for all CAZymes in the local database, facilitating updated annotations, and analyses at all levels of phylogeny, across arbitrary sets of CAZymes for which a source genome can be identified.

**Fig. 4.**
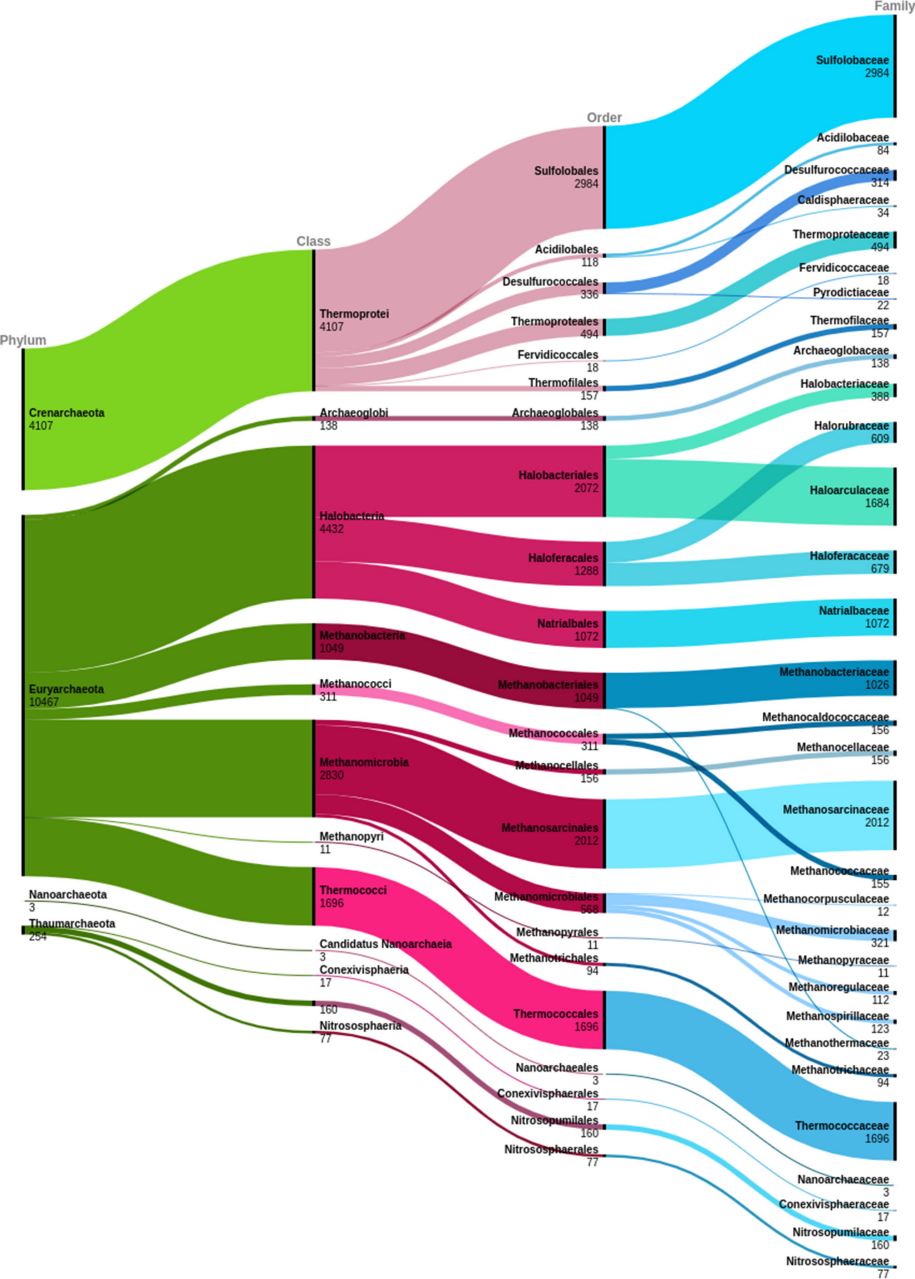
Alluvial diagram showing the count of CAZy records at each level of the NCBI archaeal taxonomy, from phylum to family. Incomplete (including ‘artificial sequences’ and ‘environmental samples’) and *Candidatus* lineages are excluded.

To demonstrate this extended capability we summarize visually the complete taxonomic representation of all archaeal CAZymes in CAZy as an alluvial plot (Fig. 4). We exclude proteins belonging to organisms assigned *Candidatus* status, or that are catalogued in CAZy but no longer present in NCBI due to withdrawal or suppression of records. This and similar analyses to identify differential representation of arbitrary taxa in CAZy cannot currently be performed via the CAZy web service. A similar dataset for all archaeal CAZymes (including incomplete lineages and organisms assigned *Candidatus* status) reporting to genus level is provided in additional file 3 (, Fig. S2), again excluding proteins that are catalogued in CAZy, but no longer present in NCBI due to record withdrawal or suppression.

By cross-referencing these data against the number of CAZymes listed per archaeal species name, we find that most archaeal CAZymes in CAZy derive from the phyla *Euryachaeota* and *Crenarchaeota *. Other archaeal phyla appear to be under-represented in CAZy, especially the phylum *Nanoarchaeota* which, despite over 250 sequenced genomes being available (May 2022), is represented by only six CAZymes in CAZy. Across the kingdom Archaea as a whole, at least one CAZyme was listed for every Archaeal family in the NCBI Taxonomy database, except for *Conexivisphaeraceae* and *Nanoarchaeaceae*. However, 35.3 % of Archaeal genera in the NCBI taxonomy were not represented in CAZy.

We find that CAZyme coverage within archaeal lineages is also uneven. Within *Crenarchaeota *, the majority of CAZymes (approximately 75 %) derive from the family *Sulfolobaceae *. The orders *Thermoproteales *, *Thermofilales* and *Desulfurococcales* are represented in CAZy approximately proportionately to the number of corresponding genomes available at NCBI. However, assuming that 1–5 % of coding sequences in a genome encode CAZymes [35], we find that the remaining orders *Acidilobales* (27 assemblies) and *Fervidiocaccales* (nine assemblies) are under-represented, having only 81 and 18 CAZymes listed in CAZy, respectively. Given the expected potential for novel biochemistries in extremophiles, these orders may represent a good opportunity for mining novel CAZymes, and this general approach could be a useful guide towards exploring CAZyme space more effectively.

### The PL20 CAZy family contains significant sequence diversity

PLs catalyse non-hydrolytic cleavage of glycosidic bonds and are exploited industrially for degrading biomass, for example in biofuel production [6, 36]. CAZy family PL20 comprises 54 β−1,4-glucuronan lyases (EC 4.2.2.14), 22 deriving from bacteria and 32 from eukaryotes, that cleave a β−1,4 linkage in the water-soluble homopolysaccharide polyglucuronate through β-elimination [37, 38]. Only one member of the PL20 family, glucuronan lyase A (gluc ly A) from *Trichoderma reesei* NBRC 31329 (BAG80639.1), has been functionally and structurally characterized in the literature to our knowledge (as of January 2022). This single characterized representative is the main basis for functional annotation transfer on the grounds of common membership of PL20.

**Fig. 5.**
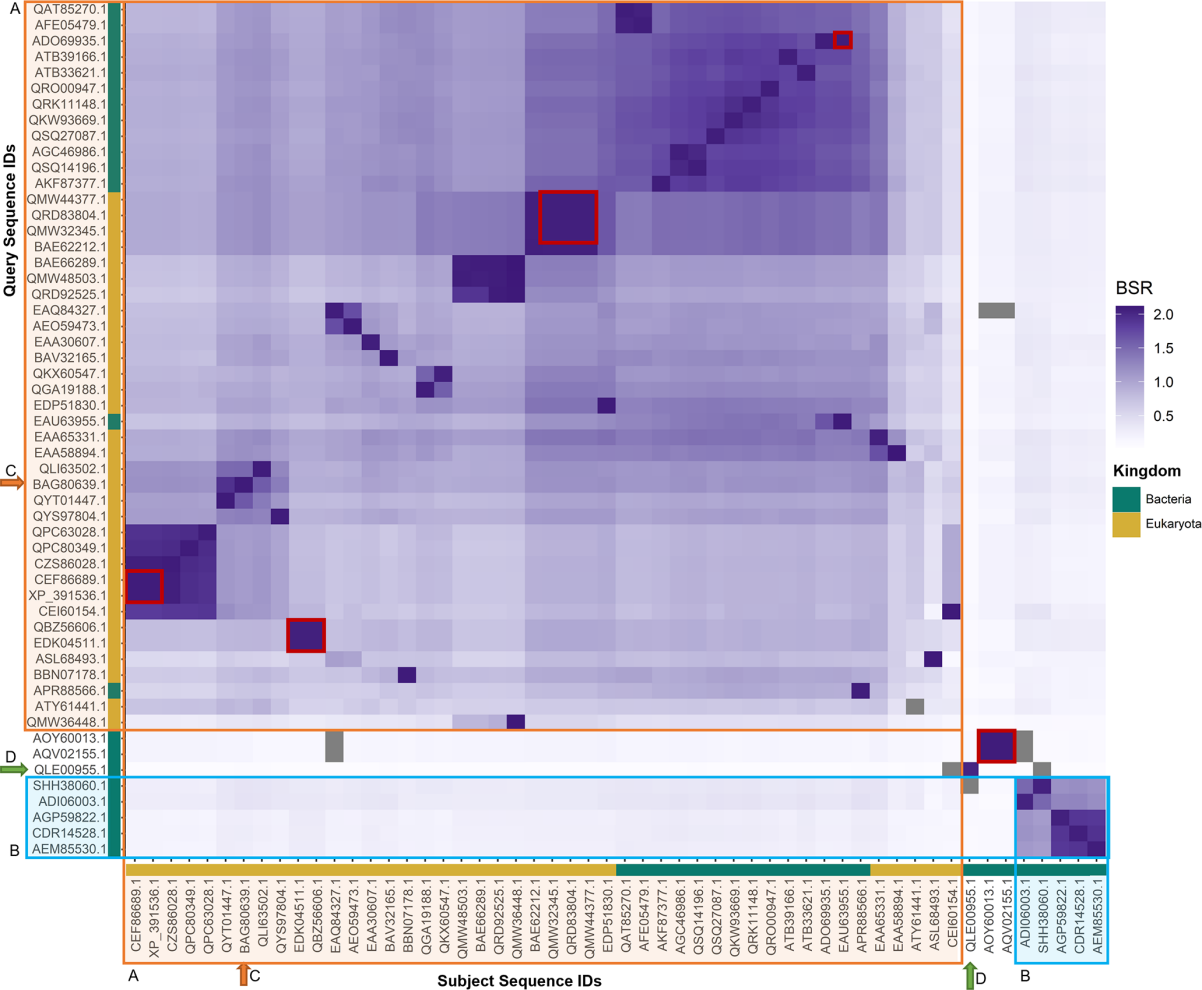
A heatmap representing BLASTP+ Score Ratios (BSRs) for CAZy family PL20 protein sequence pairwise alignments; colour intensity is proportional to BSR (more intense means proteins are more similar). The axis colour bars indicate bacterial (green) and eukaryotic (gold) CAZymes. An apparently canonical glucuronan lyase group is indicated as group A (orange outline). Group B (blue outline) contains only PL20 sequences derived from *Streptomycetes*. The only protein to be functionally and structurally characterized in the literature is glucuronan lyase A from *Trichoderma reesei* (BAG80639.1), which is indicated with an orange arrow [C]. The glucuronan lyase A from *Galbibacter* sp. BG1 is indicated by the green arrow [D]. Redundant protein sequences are outlined in red.

We explored sequence diversity within PL20 to investigate sequence similarity within the family, and to assess to what extent gluc ly A was likely to be representative of all PL20 members. Clustering of all PL20 protein sequences, as described in the Methods, identified seven pairs of redundant sequences (Fig. 5), including GenBank record CEF86689.1 and its corresponding RefSeq sequence XP_391536.1. This exemplifies that the number of sequence accessions in CAZy corresponding to a CAZy family is not always the same as the number of unique sequences.

Fig. 5 shows a clustering of PL20 sequences on the basis of sequence similarity as measured by BSR. Four distinct clusters are observed, where the members of any one PL20 cluster share less than 40 % pairwise protein sequence identity with members of any other cluster. This level of pairwise identity falls within or below the ‘Twilight Zone’ of protein sequence identity that is a heuristic threshold for the ability to infer common structure or function on the basis of sequence identity [39]. This raises the question of whether CAZy family PL20 in fact comprises a functionally or structurally consistent family of proteins.

The largest, ‘canonical’ group is a set of sequence-similar bacterial and eukaryotic CAZymes. This set includes gluc ly A, and shares an average of 55.4 % pairwise amino acid identity across most of their length (±14.4 % standard deviation, and mean 87.0 ±23.0 % coverage) (group A, Fig. 5).

A second group comprises eight bacterial sequences (group B, Fig. 5) that share an average of only 28.7 % pairwise identity (±12.1 % standard deviation, mean 79.0 ±18.4% coverage) with members of the ‘canonical’ group. Group B contains all PL20 CAZy records deriving from *Streptomyces *. This group can be further subdivided into a pair of sequences from *Streptomyces* sp. 3214.6 and *S. bingchenggensis* BCW-1, and a group of three sequences from *S. rapamycinicus* NRRL 5491, *S. iranensis* and *S. violaceusniger* Tu 4113.

The protein HX109 (HX109_05010, GenBank QLE00955.1) from *Galbibacter* sp. BG1 (green arrow, Fig. 5) is relatively isolated within the heatmap, and shares low pairwise identity with all other PL20 sequences (BSR<0.5, mean 23.8 ±7.8% identity and 34.0 ±20.6% coverage).

A redundant pair of sequences from *Desulfococcus multivorans* (sequence IDs AOY60013.1 and AQV02155.1) also have low pairwise identity with all other PL20 sequences (28.8 ±6.3% identity and 52.9 ±22.8% coverage). gluc ly A aligns only to the C-terminal region of AOY60013.1, suggesting the possible presence of an additional structural or functional domain comprising residues 1–175. However, dbCAN predicts no CAZyme domains in AOY60013.1 (additional file 4), and a BLASTP+ query against the NCBI nr database (additional file 5, accessed September 2022) matched this sequence to a conserved lignate_lyase2 superfamily domain (to which β−1,4-glucuronan lyases belong) across the full length of the AOY60013.1 sequence.

### A candidate membrane-associated PL20 CAZyme

**Fig. 6.**
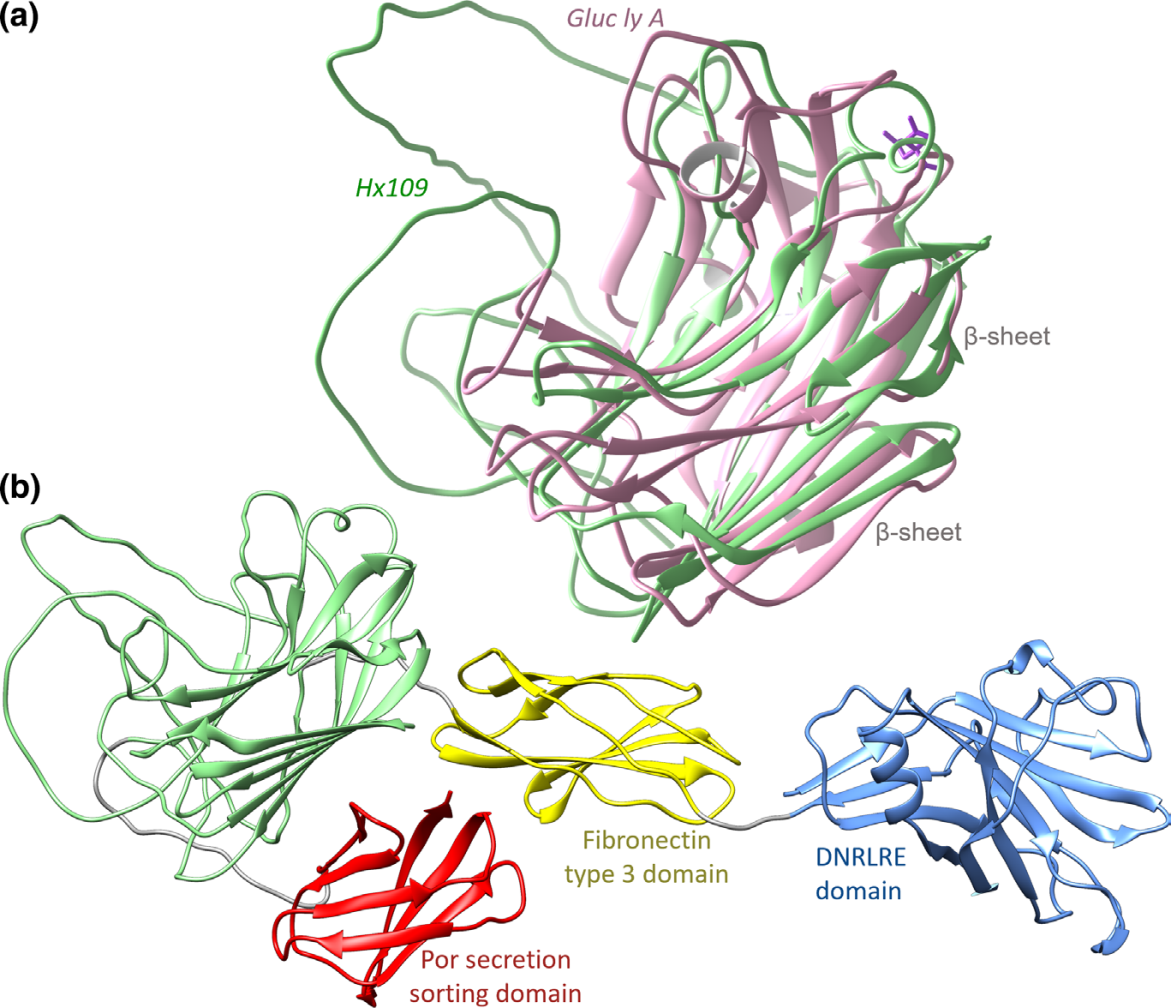
(a) The structure of HX109 as predicted by AlphaFold (green), with the structure of gluc ly A (PDB:2ZZJ; pink) superimposed onto residues 338–515 (RMSD of 1.92 Å over 137 Cα). A citric acid molecule in PDB:2ZZJ is shown in purple. (b) The complete predicted structure of HX109, which comprises, from the N- to C-termini: DNRLRE domain (blue), fibronectin type 3 domain (yellow), PL20 domain (green), and the por secretion sorting domain (red).

To explore whether HX109, which also shares low pairwise sequence identity with all other PL20 family members, might share a similar fold with gluc ly A despite being below the usual ‘Twilight Zone’ threshold for structural similarity (21.29 % pairwise sequence identity), we superimposed the gluc ly A structure (PDB:2ZZJ) [38] onto a structure predicted by AlphaFold for HX109. The AlphaFold-predicted HX109 structure possesses a similar fold to the canonical PL20 fold of gluc ly A: a β-jelly roll fold formed from two short α-helices and two anti-parallel β-sheets (Fig. 6(a)). However, the calcium ion and citric acid binding residues in gluc ly A were not conserved in the predicted HX109 structure (additional file 6; (Supplementary Material 1, Fig. S3(a,b)). The optimal structural alignment obtained by superimposing the PDB:2ZZJ structure onto HX109 PL20 domain residues 338–515 has an RMSD of 1.92 Å over 137 alpha carbons (Cα).

The complete predicted structure of HX109 is larger than a single PL20 domain. Rather, it appears to be composed of four domains, each composed of a pair of β-sheets (Fig. 6b). The global placement of each domain relative to the others is predicted with low confidence by AlphaFold (additional file 7) and so the complete conformation of the protein structure remains uncertain. However, a BLASTP+ query of the full-length HX109 protein sequence against the NCBI nr database (additional file 8) identified: (i) a conserved alginate lyase_2 superfamily (PFAM14099) domain that is also present in gluc ly A; (ii) a C-terminal por secretion sorting domain (Pfam:PF18962); and (iii) a fibronectin type 3 domain (Pfam:PF00041), which is found in many extracellular bacterial CAZymes [40, 41]. A conserved N-terminal DNRLRE domain [NCBI Conserved Domains Database (CDD):NF033679], typically involved in cell wall function and structural organization, was also identified [42]. DeepTMHMM [28] did not predict any transmembrane domain in HX109 (see Methods, additional file 9). SignalP predicted the presence of a signal sequence associated with a general secretion pathway. Taken together the signal peptide, along with the por, fibronectin and DNRLRE domains suggest that HX109 may be secreted and might perhaps be membrane-associated. The BLASTP+ query of HX109 against all other CAZy PL20 sequences did not identify any of these indicators of potential membrane association, and so HX109 may be the only currently known candidate membrane-associated PL20 CAZyme.

### Identification of structurally characterized carbohydrate esterases

Automated integration of sequence and structural data from databases such as UniProt and RCSB extends cazy_webscraper’s scope beyond simple replication of CAZy, and enables enhanced functional analysis of these proteins. To demonstrate this, we assessed the degree of structural characterization of CE families by retrieving from UniProt all RCSB PDB IDs for the 104,680 CAZymes catalogued under the 20 CE families defined by CAZy. Obtaining these data took 3 min 10 s (± 5.23 s, three replicates). The number of CAZymes in each CE family associated with at least one PDB ID was retrieved as described in the Methods (elapsed time: 15 min 41 s, ±37 s, three replicates, Table 3).

**Table 3.** The number of CAZymes in each CE family (January 2022), and the number of CAZymes in each family associated with at least one PDB ID in UniProt (April 2022)

Family	Total CAZymes	CAZymes with PDB IDs in UniProt
CE_0	2960	7
CE_1	5026	6
CE_2	709	4
CE_3	600	3
CE_4	34 194	27
CE_5	4195	10
CE_6	453	1
CE_7	2664	5
CE_8	8927	7
CE_9	19 773	7
CE_10	0	0
CE_11	13 842	4
CE_12	3452	3
CE_13	484	0
CE_14	6487	6
CE_15	583	8
CE_16	189	0
CE_17	18	1
CE_18	37	1
CE_19	251	1
Total	104 844	101

Sixteen CE proteins that were not annotated in CAZy as being structurally characterized were identified as having a solved structure by retrieving PDB IDs from their corresponding UniProt entry. However, 31 proteins annotated in CAZy as structurally characterized did not have an associated PDB ID recorded in their corresponding UniProt entry.

We find that CE families vary in the number of available representative structures. This information may guide strategic efforts to improve structural characterization of families that are unrepresented or under-represented in the RSCB PDB. For example, no PDB IDs were retrieved for CE families 13 and 16, and only one CAZyme in each of the CE families 6, 17, 18 and 19 has been structurally characterized to date. These families may be worthwhile targets for systematic structure determination.

### Identification of a possible novel CE domain structural variant

All members of a CAZy family are generally expected to share a common structural fold [43]. CAZy families with no structural representatives at all may be the most promising candidates for novel structure characterization. However, protein sequences that share less than 40 % identity cannot be assumed to share a highly conserved backbone structure [39]. We have already established that there is significant sequence diversity in the PL20 CAZy family and, where a CAZy family can be subdivided into subgroups of proteins sharing more than 40 % identity within, but less than 40 % identity between, subgroups it may be that a subgroup potentially possesses a structural fold that varies from the characteristic structure for the family as a whole. This represents a second, distinct group of strategic targets where structure determination may reveal novel folds.

To investigate potential structural variation in these low sequence identity groups, we used cazy_webscraper to retrieve all 251 protein sequences for CE19 family members from the NCBI Protein database. We clustered these sequences at a threshold of 40 % identity and 80 % coverage using MMSeqs2, and identified clusters containing no structurally characterized proteins (additional file 10).

We noted that a predicted xylan esterase from *Luteitalea* sp. TBR-22 (TBR22) comprising 634 residues, TBR22_41900 (TBR22, BCS34995.1), did not cluster with any other CE19 proteins by amino acid sequence. We used TBR22 as a BLASTP+ query against the CE19 family, observing no match with amino acid identity above 32.2 % (additional file 11). All such BLASTP+ alignments matched residues 1–380 in TBR22, implying the presence of a conserved N-terminal CE19 domain. However, few CE19 proteins matched against TBR22 residues 380–634. This implied that TBR22 contains a conserved CE19 domain (residues 1–380), and a second domain potentially with unknown function (residues 380–634).

However, a single predicted acetylxylan esterase (D6B99_08585, AYD47656.1) was observed to align to TBR22 residues 57–615, corresponding to residues 190–779 in D6B99_08585. dbCAN predicts that D6B99_08585 residues 120–457 comprise a CE19 domain, but identifies no CAZy family domain for residues 458–779 (additional file 12). dbCAN did not, however, predict a CAZy family domain in TBR22.

Querying the TBR22 C-terminal domain (residues 380–634) against the NCBI nr database using BLASTP+ (see Methods) identified no putative conserved domains according to the Conserved Domain Database (CDD), but primarily returned hits against acetylxylan esterases from *Acidobacteria* (additional file 13). Using the sequence of TBR22 residues 380–634 as a query against the PDB database also returned no matches (June 2022).

**Fig. 7.**
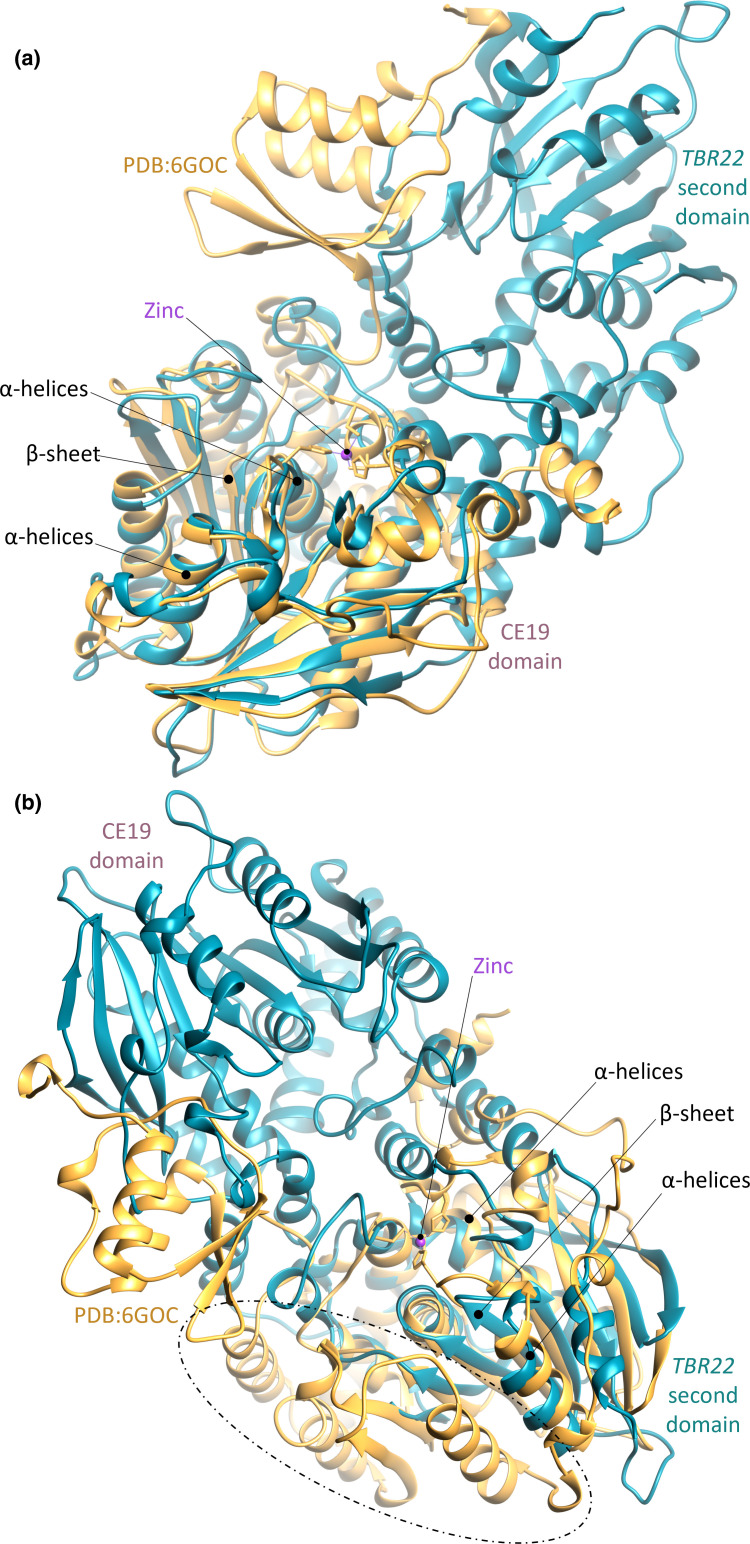
The structural fold of TBR22_41900 (TBR22) as predicted by AlphaFold, shown by secondary structure in teal. Protein structure PDB:6GOC (shown in orange) superimposed onto (a) TBR22 (RMSD 2.05 Å across 311 Cα), and (b) the second TBR22 domain (RMSD 2.24 Å across 200 Cα) using the Chimaera MatchMaker and Match-Align tools. The zinc ion in PDB:6GOC is shown in purple. Major differences between 6GOC and the predicted TBR22 fold are highlighted in the dashed-line oval.

We predicted a candidate protein structure for TBR22 using AlphaFold (additional file 14), and structurally superimposed the structure of PDB:6GOC (ALJ42174.1), the only CE19 structure listed in CAZy and UniProt, onto that prediction (Fig. 7(a)). 6GOC aligned well to the predicted CE19 domain (RMSD 2.05 Å across 311 Cα), indicating that 6GOC and TBR22 share a common α/β hydrolase fold, consisting of a three-layer α/β/α sandwich containing a nine-stranded β-sheet. The two histidines in the 6GOC zinc ion binding site were conserved in the TBR22 CE19 domain (additional file 6; Supplementary Material 1, Fig. S3(c)), suggesting that this feature was retained. 6GOC was aligned onto the second TBR22 domain (RMSD 2.24 Å across 200 Cα) (Fig. 7(b)). The second predicted TBR22 domain displays an α–β–α sandwich fold that resembles the 6GOC α/β hydrolase fold, but contains a seven-stranded, rather than a nine-stranded, β-sheet, and has two fewer α-helices below and one fewer α-helix above the β-sheet sandwich. Additionally, the zinc ion binding site in 6GOC was not conserved in the second predicted TBR22 domain (additional file 6; Supplementary Material 1, Fig. S3(d)). We note that the 6GOC structure excludes 190 N-terminal residues present in the full-length ALJ42174.1 protein sequence. We cannot therefore conclusively assert that the second domain observed in TBR22 is absent in the full-length ALJ42174.1 sequence.

A BLASTP+ query of the TBR22 C-terminal domain sequence against all CE protein sequences that have at least one associated PDB accession in UniProt (additional file 15) produced no alignment that matched more than 5 % of the domain. The full-length TBR22 sequence was queried iteratively against the RCSB PDB using HHPred to identify remote protein homologues not already catalogued in CAZy that may represent the full-length and/or TBR22 C-terminal domain structural fold in RCSB PDB (additional file 16). The three highest-scoring structures (probability score and E-value were 99.5–99.7% and 4E-13 to 1.3E-15, respectively): acetyl xylan esterase 3NUZ chain C (CAH07500.1); SusD/RagB-associated esterase-like protein 3G8Y (ABR41713.1); and RNA polymerase I subunit 6RUI chain A (AAA34992.1). These were aligned against the full-length TBR22 predicted structure and against the TBR22 second domain (see Methods) (additional file 17; Supplementary Material 1, Fig. S4).

The structural alignments of 3NUZ and 3G8Y against full-length TBR22 (RMSD 1.85 Å across 275 Cα and RMSD 1.95 Å across 278 Cα, respectively) and the second TBR22 domain (RMSD 1.89 Å across 209 Cα and RMSD 1.97 Å across 215 Cα, respectively) are similar to those observed with 6GOC. 6RUI shows low structural similarity to full-length TBR22 (RMSD 2.23 Å across 231 Cα) and the TBR22 C-terminal domain (RMSD 2.35 Å across 174 Cα) (additional file 17; Supplementary Material 1, Fig. S4). We therefore propose that the second TBR22 domain may potentially be a previously undescribed variant of the structural fold conserved in other CE19 enzymes.

### Prediction of a candidate novel CAZyme domain architecture – CE12:CBM35:PL11

We identified 174 clusters of CE12 protein sequences using MMSeqs (additional file 18). Only two of these clusters contained sequences with a structurally characterized CE12 domain (we excluded ABN54336.1, which is associated with PDB ID 2W1W, as this structure represents only a CBM domain). We then attempted to determine if these two structurally characterized proteins (CAA61858.1, CAB15948.2) were likely to be representative for all CE12 family members. Eight of the 172 clusters with no characterized structure, comprising in total 50 sequences, contain proteins annotated with both CE12 and PL11_1 subfamily domains. Two enzymes in this group also include a CBM domain: QNU65479.1 contains a CBM35 domain and QZD56848.1 a CBM2 domain. To our knowledge none of the enzymes containing both CE12 and PL11_1 domains have yet been functionally and/or structurally characterized, so it was unclear to what degree the typical CE12 and PL11 activities, catalytic mechanisms and structures were conserved in these enzymes.

We arbitrarily chose the *Metabacillus* sp. KUDC1714 CAZyme HUW50_16055 (HUW50, QNF30995.1) as a basis to explore whether a fold with CE12 and PL11 domains may already be structurally represented in the RCSB PDB database. The domain ranges of the CE12 and PL11 domains were annotated as described in the Methods, which resulted in the unexpected prediction of an additional CBM35 domain in this protein (although we note that CBM35 domains are observed with other CE12 proteins). The CE12 domain was annotated as residues 22–440, the CBM35 domain as residues 440–644, and the PL11 domain as residues 806–1423. The structural fold for each domain was predicted independently using AlphaFold (additional file 19, Fig. 8).

**Fig. 8.**
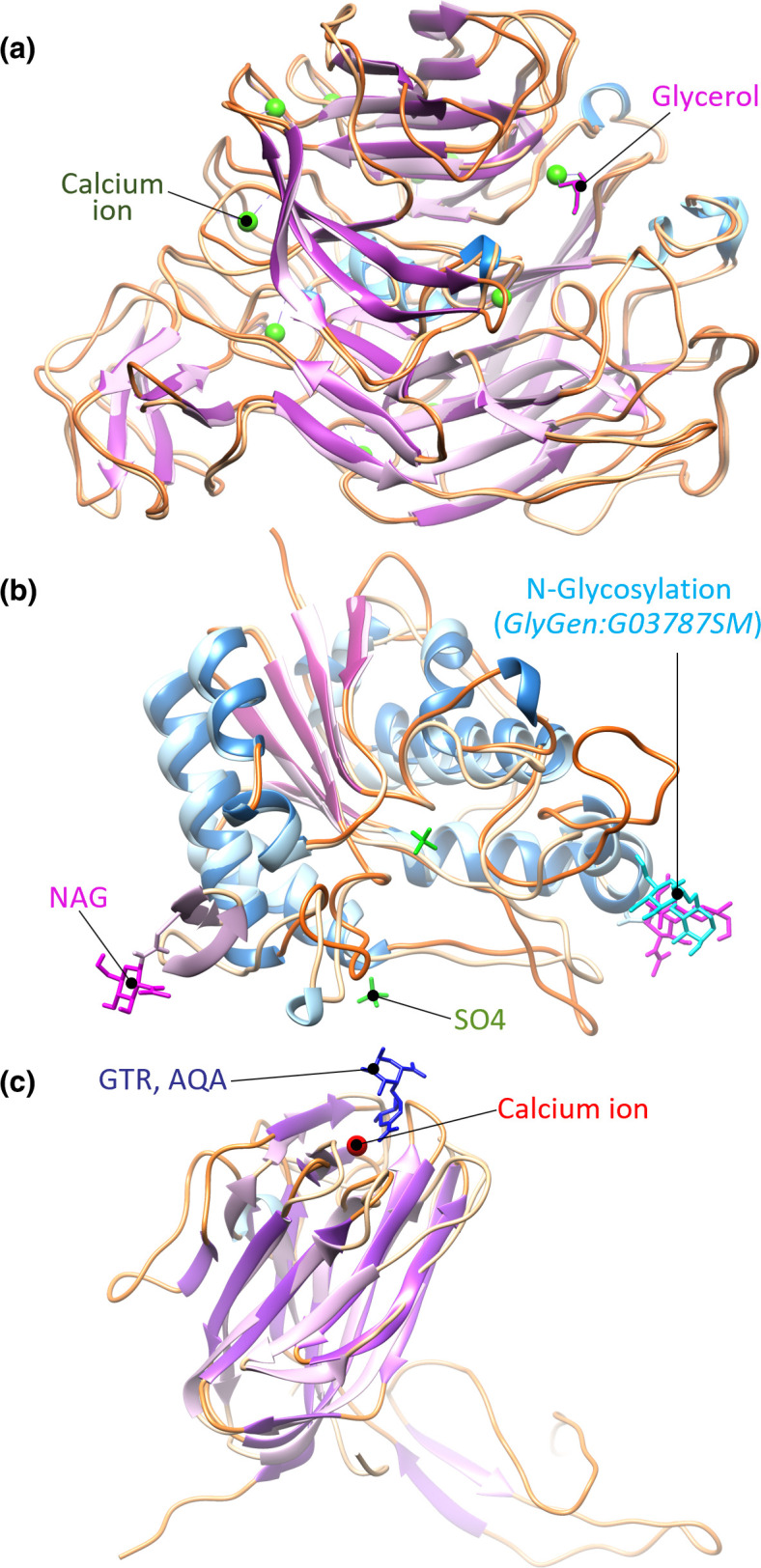
AlphaFold-predicted structures for each of the three CAZyme domains of HUW50_16055 (HUW50, NCBI QNF30995.1), showing the secondary structure. α-Helices are shown in blue, β-sheets in purple, the loop regions in orange and calcium ions in dark green. The predicted structure is shown at greater opacity. (a) The predicted protein structure of Huw50 PL11 domain superimposed onto PDB:4CAG with the 4CAG glycerol ligand shown in pink. (b) The predicted CE12 domain structure superimposed onto PDB:1DEO, with the 1DEO N-linked acetylglucosamine (NAG) shown in pink and the N-glycosylation (GlyGen:G03787SM) shown in light blue, and the two sulphate ions (SO4) shown in green. (c) The predicted CBM35 domain structure superimposed onto PDB:2W47 with the ligand 4-deoxy-β-l-threo-hex-4-enopyranuronic acid-[1,4]-β-d-galactopyranuronic acid (GTR, AQA) shown in dark blue.

The predicted PL11 domain structure in HUW50_16055 forms a characteristic eight-bladed β-sheet propeller structure (Fig. 8(a)) [44–46] similar to the PL11 CAZyme RGL11 from *Bacillus licheniformis* (PDB: 4CAG) (RMSD 0.72 Å across 580 Cα) [46]. Additionally, the ten calcium ion binding sites in RGL11 are conserved in HUW50 (additional file 6; Supplementary Material 1, Fig. S3(e)). The predicted fold of the HUW50 CE12 domain forms the characteristic five-strand β-sheet sandwich between α-helices [47, 48] and aligns well to the CE12 enzyme RGAE from *Aspergillus oryzae* (PDB: 1DEO) [47] (RMSD 1.54 Å over 199 Cα) (Fig. 8(b)). The RGAE catalytic triad, composed of a (nucleophilic) Ser, Asp and His, is conserved in the HUW50 prediction (additional file 6; Supplementary Material 1, Fig. S3(f)), suggesting the enzyme may be catalytically active. However, the N-linked glycosylation site, comprising a short α-helix and small β-sheet in RGAE, was not predicted for HUW50. The CBM35 domain of CTHE_3141 from *Acetivibrio thermocellus* (PDB: 2W47) aligns to the predicted structure of the HUW50 CBM35 domain (RMSD 2.31 Å across 105 Cα) (Fig. 8(c)), implying that HUW50 does contain a CBM35 domain not yet annotated in its CAZy record. This highlights the difficulty of annotation for multi-domain CAZymes, and that some classifications missed in the CAZy database may be identifiable if automated annotation were to be used.

## Discussion

CAZy is a valuable resource that provides a curated, sequence-based classification of carbohydrate processing enzymes. It enhances the community’s ability to develop predictors of enzyme function, catalytic mechanism and structure, so is an ideal starting point for bioinformatic mining and cataloguing of the many carbohydrate-processing enzymes revealed by the rapidly increasing corpus of publicly available sequence data. Using CAZy for predictive tasks generally requires automated data processing, but the community’s ability to systematically use, or analyse, the data in CAZy is limited. This is in part due to the lack of an API for complex queries in the CAZy database, which is understandable given funding and resource constraints [6]. Even with such an interface, users would need themselves to integrate CAZy data with sequence, structure and other datasets, which presupposes some ability with data analysis and programming. cazy_webscraper provides a programmatic interface to the downloadable CAZy data, but also provides tools for analysing it that require no prior programming knowledge (by which we mean that database construction and CAZy analysis can be performed using only terminal commands, and without development and coding of programming scripts or packages). This makes the dataset much more accessible and facilitates programming-free automated analysis for common tasks, including the use and extension of CAZy with data from public taxonomic, genomic and structure databases, with no additional burden on the CAZy resource beyond obtaining the publicly available compressed database download.

CAZy does not store or directly provide sequence or structure data for its records. These data are instead linked from the CAZy record. This can place a non-trivial programming or database integration requirement on users of workflows employing CAZy in combination with other databases. cazy_webscraper provides commands for actions simplifying this integration, including: (i) retrieving and locally storing sequence data for CAZy records; (ii) eliminating redundant records; (iii) allowing filtering and querying with user-defined criteria, to reduce the dataset to arbitrary groups of interest; and (iv) making filtered sequence sets available in standard formats for downstream alignment. cazy_webscraper facilitates integration of CAZy data with common bioinformatic analyses (clustering, phylogenetics, positive selection, CAZyme prediction, etc.).

We show that, by making sequence and structural data for CAZy records more conveniently available, cazy_webscraper helps identify CAZymes of strategic interest for further investigation. These enzymes may be potentially unrepresentative of their assigned CAZy classes or families, or otherwise of interest for functional or structural characterization, potentially facilitating discovery of previously undisclosed CAZyme diversity (Fig. 5). In addition, this enables sequence–structure–function analyses such as mapping of conservation or correlated changes onto structure, potentially improving prediction of conserved sites and functional characterization. In this paper we identify: (i) potentially the first example of a membrane-associated PL20 enzyme (from *Galbibacter *); (ii) a distinct cluster of *Streptomyces* PL20 sequences with minimal sequence similarity to other bacterial PL20s; (iii) a *Desulfococcus multivorans* protein with minimal similarity to any other PL20 record in CAZy; (iv) a potential new CE12 fold variant; and (v) a candidate novel CE12:CBM35:PL11 domain architecture. These preliminary analyses suggest that a wealth of novel CAZyme features may await discovery, given the ability to automatically interrogate the CAZy dataset.

cazy_webscraper is not the only tool that integrates data from the CAZy database into a data model, but we believe its functionality either complements or extends those currently available tools. A tabular comparison of operations for a selection of common tools is presented in additional file 30 (Supplementary Material 1, Table S1). As one example, CAZyme classifiers such as dbCAN and CUPP use data from the CAZy database to train classification algorithms, implicitly integrating CAZy family annotations into their data models [33, 36]. The primary function of CAZyme classifiers such as dbCAN and CUPP is to predict CAZyme annotations for genomic or other sequences provided by the user [33]. CUPP and dbCAN can be operated locally and via their respective webservers but it is not possible, to our understanding, to modify the training sets for the online tools. If one wishes to analyse newly predicted CAZymes in the context of a larger database containing user-defined sequences, this must be done with a local installation of the package and a custom-compiled database. The database compilation role can be managed by cazy_webscraper, which integrates predicted CAZyme sequences and the annotations into a reproducible local CAZyme database. In this way cazy_webscraper complements prediction tools, including dbCAN and CUPP.

The CUPP and dbCAN servers both provide the ability to browse and download, in whole or in part, the underlying CUPP and dbCAN databases, which include CAZy classifications [33, 36]. To that extent, both tools perform a similar task to cazy_webscraper. In addition to CAZy classifications, the CUPP database includes CUPP-predicted CAZyme classifications. Other functional prediction approaches, not focused on but still implicitly predicting CAZymes (e.g. by generating clustered orthologous groups as in eggNOG and COG), also effectively generate novel CAZyme classifications. However, to our knowledge, the CUPP, dbCAN and more general functional prediction databases do not integrate on-demand sequence or structural data for downstream analysis into their online datasets for subsequent prediction or analysis. cazy_webscraper goes beyond simple collection of CAZy data to integrate additional data sources, extending beyond the capabilities of the CAZyme-targeted CUPP and dbCAN servers in particular (several of these actions with commands are represented in Table 1). Moreover, dbCAN and CUPP provide their downloaded databases as flat files which require programming, or a tool such as cazy_webscraper, in order to construct a queryable data model. The CUPP server does enable filtering and selection of sequences from their precompiled database using simple checkboxes prior to download, but the filtering operations are manual *via* the browser interface, and restricted in scope relative to cazy_webscraper. It is not, for instance, possible to simultaneously filter the CUPP database on taxonomy and CAZy class via the browser interface. cazy_webscraper thus extends the user’s capabilities for data analysis beyond that possible in these interfaces.

The central operation of cazy_webscraper is construction of a queryable data model, i.e. an SQLite3 database. Both CUPP and dbCAN databases rightly focus on the predictions made by their corresponding tools. cazy_webscraper provides a means of extending these, and other tools, by integrating predictions made by multiple methods, enabling their direct comparison and/or more complex manual or automated queries of the imported database(s). Extending the accessibility of this operation, including the ability to expand the local CAZyme database through inclusion of predicted CAZyme annotations, without the need for programming, is in the future roadmap for cazy_webscraper.

An alternative tool, SACCHARIS, is a pipeline that streamlines selection of uncharacterized sequences for experimental exploration. SACCHARIS is targeted specifically towards detecting new CAZyme or CBM specificity from families in the CAZy database or in user-defined datasets, for which it may also perform CAZyme class prediction [49]. Streamlining selection of uncharacterized sequences was also a motivating factor in the development of cazy_webscraper. However, a key distinction is that SACCHARIS takes a database (or collection of sequences) as input for a specified analysis encoded in SACCHARIS, whereas cazy_webscraper is designed to make it easier for scientists to compile their own databases from CAZy and other resources and clean and analyse those data, for use with tools such as SACCHARIS. Thus cazy_webscraper complements SACCHARIS in that it can compile appropriate input data for the pipeline, a function not represented in SACCHARIS itself (in the SACCHARIS announcement, CAZy data were obtained using a separate ‘in-house program’) [49]. Similarly, we could have used SACCHARIS in our downstream analyses, but chose to illustrate aspects of the data that are not revealed by the SACCHARIS pipeline, instead demonstrating compilation of a customized local CAZyme database integrated into bespoke downstream analytical pipelines guided by the requirements of the user.

In this paper we demonstrate the application of cazy_webscraper, complementary to and extending tools and databases including those discussed above, to download the complete CAZy database, compile it into a local SQLite database, and enhance it with additional information from public datasets, enabling reproducible, flexible and detailed interrogation of the CAZy database (Table 1). Download and analysis of the complete CAZy database with cazy_webscraper generates otherwise unavailable summary information about CAZy’s composition and comprehensiveness that is not evident using the CAZy web interface alone (Fig. 3). We note that CAZy is constrained by data inclusion policies; for example, a maximum of 60 strains per species is considered for any CAZy family. This caps the sequence diversity that can be represented in the database and limits the value of CAZy for within-species diversity analyses and pan-genomic studies [6]. Extending CAZy to the necessary scope for these analyses requires the use of tools such as cazy_webscraper to integrate data from other resources. By conducting large-scale predictions using tools such as dbCAN (or importing their public databases) and incorporating the output into cazy_webscraper’s local database, the relatively restricted CAZy dataset can be extended to facilitate more expansive and more taxonomically targeted studies.

Analysis of the complete CAZy dataset using cazy_webscraper enables data cleaning to refine the dataset for other purposes, such as generation of non-redundant training sets for machine learning. For example, our survey of family PL20 demonstrates that CAZy contains redundant protein sequences that should be removed before incorporation into training sets for predictive tools. In particular, we identify cases where both GenBank and RefSeq accessions are catalogued as primary records for the same sequence, implying redundant representation of the same protein sequence under two accessions. Automated identification of redundant accessions also benefits phylogenetic reconstruction. For example, some RefSeq accessions included represent Identical Protein Groups (IPGs) that have redundant amino acid sequences in several organisms but may correspond to multiple proteins with distinct nucleotide coding sequences that can be recovered from public databases. We have shown how CAZy summary data identify opportunities for targeted functional and structural characterization of under-studied groups of enzymes. This assists more effective sampling, and expansion of our understanding of CAZyme diversity across taxa (Fig. 4).

### Extending CAZy’s taxonomic data

The CAZy database uses taxonomic assignments retrieved from NCBI. Unfortunately, taxonomic opinions are not always consistent between reference taxonomies, and may be revised over time within a single taxonomic resource [12]. At NCBI taxonomic assignments are usually provided upon deposition by the sequence submitter but are not always updated in a timely manner to reflect revised understanding or a change of opinion. This observation was a motivation for construction of the widely used GTDB which aims to provide up-to-date genome-based bacterial and archaeal taxonomies [12]. cazy_webscraper can integrate assigned taxonomy from either or both of NCBI Taxonomy and GTDB, providing users with a choice and comparator of taxonomic references.

For some downstream analyses, including building sequence-based CAZyme classifiers, it is desirable to account for bias in the input training sequence set, such as over-representation of a particular taxonomic group. We can identify the probable influence of sampling bias in CAZy, but so as long as taxa are unevenly sampled in the underlying sequence databases from which CAZy is constructed this bias will remain difficult to rectify. Using cazy_webscraper we show that the overall distribution of CAZymes by kingdom reflects NCBI’s known sampling bias towards bacterial genomes [for example, the bacterium with the greatest number of RefSeq assemblies – 171 542 - is *Escherichia coli *, but the most-sequenced eukaryote, *Homo sapiens*, has only 1268 (July 2022)]. However, we find that CAZy class and family distribution at kingdom level is not only driven by NCBI database composition but may also reflect biologically significant differences. Specifically, we show that the AA class is dominated by eukaryotic sequences, consistent with the CAZy curators’ own observation that members of the AA class are strongly biased towards fungal enzymes [50] (Fig. 3), and also that many families of archaea are under-represented in terms of their coverage in CAZy compared to NCBI (Fig. 4). cazy_webscraper provides a mechanism by which under-sampled taxa may be identified, to help direct future strategic efforts in characterizing CAZyme diversity. To facilitate this, an automated taxonomy survey of the CAZy dataset is in our development roadmap for cazy_webscraper.

### Extending structural information for CAZy records

We show how cazy_webscraper can extend the core CAZy dataset to include local annotation, sequence, taxonomic and structural data from the NCBI, UniProt, GTDB and RCSB PDB databases, so it can be integrated more easily into extensive sequence, functional, structural and evolutionary studies. The CAZy website lists PDB IDs of CAZymes represented in the RCSB PDB database, but these must be identified and retrieved manually by the user. cazy_webscraper gathers PDB IDs from the corresponding UniProt record for each CAZyme in an automated manner. We find that identifying structural data via UniProt identifies additional PDB entries not currently listed in CAZy. For example, we find that nearly 20 % of all CE class members associated with a PDB ID in UniProt are not annotated as structurally characterized in CAZy. We also find instances where CAZy lists PDB IDs for a record that are not annotated in the corresponding UniProt entry. We were thus able to use cazy_webscraper to consolidate and clean structural information provided in the CAZy dataset.

### Identifying potentially industrially exploitable enzymes

CE enzymes remove methyl or acetyl groups that shield the polysaccharide backbone from degradation by GHs and PLs. Consequently, many CEs are industrially exploited to increase the surface area accessible to polysaccharide backbone-degrading enzymes, thus increasing the efficiency of polysaccharide degradation [51]. A common substrate for enzymes in several CE families is rhamnogalacturonan (RG), a structurally complex glycan that contains 13 unique monosaccharides and 21 distinct glycosidic linkages, nearly all of which require a bespoke enzyme for their cleavage [52]. RG is highly abundant in plant cell walls and a rich source of monosaccharides for biofuel production [52, 53]. It is possible that by mining CAZyme data it could be possible to extend the range of known CE enzyme specificities better to cover the range of naturally occurring RG types, and enhance the efficiency of degradation for industrial purposes, or to improve our understanding of sequence–structure–function relationships sufficiently to aid engineering of these enzymes.

Using cazy_webscraper we identified two CE enzymes having no representative structure in the PDB [TBR22_41900 (TBR22) from *Luteitalea* sp. TBR-22, and a CE12-PL11 enzyme (with potentially a CBM35 domain) HUW50_16055 (HUW50) from *Metabacillus* sp. KUDC1714]. These enzymes shared relatively little (<40 %) sequence identity with any other CE19 sequence in CAZy, and we suspected they might possess a novel or variant structural fold for the family. Their characterization might, therefore, extend our coverage of the known CE19 protein structure space and aid elucidation of the enzyme family’s mechanism.

The CE19 family contains a single functionally characterized member, BT1017 (AAO76124.1 [52]). BT1017 is a pectin methylesterase that removes methyl esters from rhamnogalacturonan-II (RG-II) [52, 53]. This activity facilitates complete degradation of the RG-II backbone by RG-backbone-degrading enzymes [52]. The structural folds shared by BT1017 (PDB:6GOC) and the AlphaFold-predicted structure of the first domain of TBR22 are similar, and it is possible that TBR22 could possess the same RG-II-backbone degradation behaviour as BT1017. However, the predicted structural fold of the second TBR22 domain did not show identifiable similarity with any structures in the RSCB PDB, which suggests it might play a different role or target an unknown substrate, perhaps working synergistically with the TBR22 CE19 domain. This suggests that TBR22 warrants further investigation and that CE19, and perhaps other CAZy families, harbour greater structural and functional diversity than currently suspected.

Family CE12 comprises acetylesterases that target the plant cell wall polysaccharides pectin, RG and xylan (EC 3.1.1.-). Several characterized CE12 enzymes display synergistic activity resulting in an increased rate of polysaccharide degradation. For example, synergy between the RG-lyase YesT and a xylanase significantly increases the rate of degradation of acetylated xylan [54]. CAZy family PL11 represents rhamnogalacturonan lyases (EC 4.2.2.23 and EC 4.2.2.24) that degrade the RG backbone via β-elimination [45]. The synergistic activity between an RG-acetylesterase and the RG backbone-degrading enzymes RGase A and B is known to significantly increase the rate of RG degradation [55]. We predict that HUW50 also contains a CBM35 domain, examples of which are known to target RG [56]. We therefore speculate that the HUW50 CE12-PL11 protein contains a PL11 domain that acts synergistically with CE12 and a CBM in a novel synergistically linked domain composition to more efficiently degrade RG.

HUW50 is only one example of 50 candidate multidomain CE12:PL11 proteins we identify for the first time in our study. To our knowledge no protein structures in the RCSB PDB represent the global structure of these enzymes. The synergistic activity we speculatively propose has not been directly experimentally determined, nor have their substrate(s) been fully characterized. However, the family activities listed in CAZy imply that these enzymes may degrade plant cell-wall polysaccharides, and that investigation of their properties could be of biotechnological interest.

In conclusion, cazy_webscraper automates retrieval and integration of user-specified datasets from the CAZy, NCBI, GTDB, UniProt and RCSB PDB databases to create a local CAZyme database of proteomic, taxonomic and structural data. The commands used to create the dataset can be provided in a YAML configuration file and shared or re-run for reproducible creation and update of datasets. The compilation of CAZy records into a comprehensively logged SQLite3 database enhances transparency, reproducibility, shareability and replicability of analyses. Thus, cazy_webscraper facilitates mining an extended CAZy dataset via bioinformatic discovery workflows, enabling identification of enzyme candidates of interest for novel functional and/or structural characterization. We expect this to be of particular use for industrial biotechnology applications, and in particular biofuel production.

## Supplementary Data

Supplementary material 1
