# Supplementary material for: cazy_webscraper: local compilation and interrogation of comprehensive CAZyme datasets
Source: Microb Genom. 2023 Aug 14;9(8):mgen001086. doi: 10.1099/mgen.0.001086 (PMC10483417; doi:10.1099/mgen.0.001086)
Supplement: Supplementary material 1 [file mgen-9-1086-s001.pdf]

cazy_webscraper: local compilation and interrogation of comprehensive CAZyme datasets

1.1 Author names

Emma Elizabeth Mary Hobbs https://orcid.org/0000-0002-8470-4739 [A, B, C]
Tracey Maureen Gloster https://orcid.org/0000-0003-4692-2222 [A]
Leighton Pritchard http://orcid.org/0000-0002-8392-2822 [B]

1.2 Affiliation(s)

A. School of Biology and Biomedical Sciences Research Complex, University of St Andrews, North Haugh, St Andrews, Fife, KY16 9ST, UK
B. Strathclyde Institute of Pharmacy and Biomedical Sciences, University of Strathclyde, Glasgow, G4
C. Cell and Molecular Sciences, James Hutton Institute, Invergowrie, Dundee, DD2 5DA, UK

1.3 Corresponding author and email address

Tracey Gloster – tmg@st-andrews.ac.uk

1.4 Keywords

CAZy, CAZymes, Database, Lignocellulose, Software, Carbohydrate Active enZymes

1.5 Repositories

Project home page: https://hobnobmancer.github.io/cazy_webscraper/
GitHub Repository: https://github.com/HobnobMancer/cazy_webscraper
Documentation: https://cazy-webscraper.readthedocs.io/
DOI: https://doi.org/10.5281/zenodo.6343936

Supplementary Figure 1. Kingdom distribution per CAZy family. The proportions and presence/absence plots of CAZymes per taxonomic kingdom in each CAZy family.

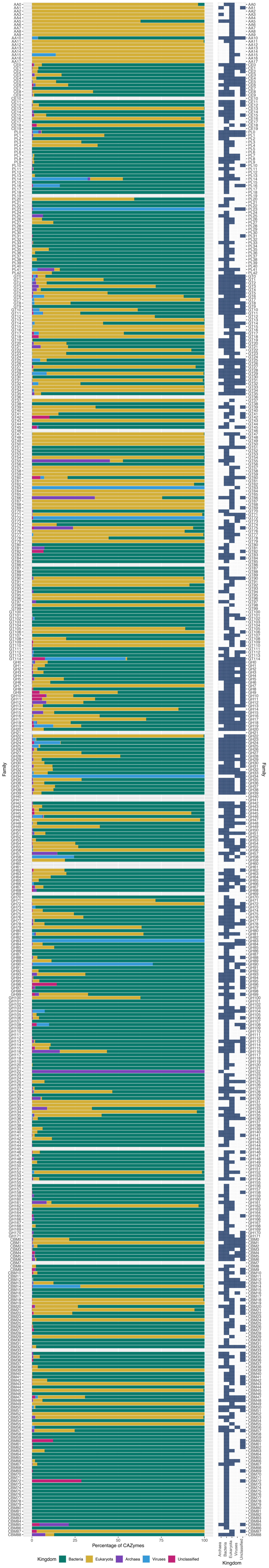

Family
Percentage of CAZymes
0
25
50
75
100
Family
Archaea
Bacteria
Eukaryota
Viruses
Unclassified
Kingdom
Kingdom
Bacteria
Eukaryota
Archaea
Viruses
Unclassified

Supplementary Figure 2. NCBI archaeal lineages. Alluvial plot for all lineages retrieved from NCBI for all archaeal CAZymes in CAZy, from kingdom to genus. Full sized figure with interactive tool tips containing annotation data is available in the online repository.

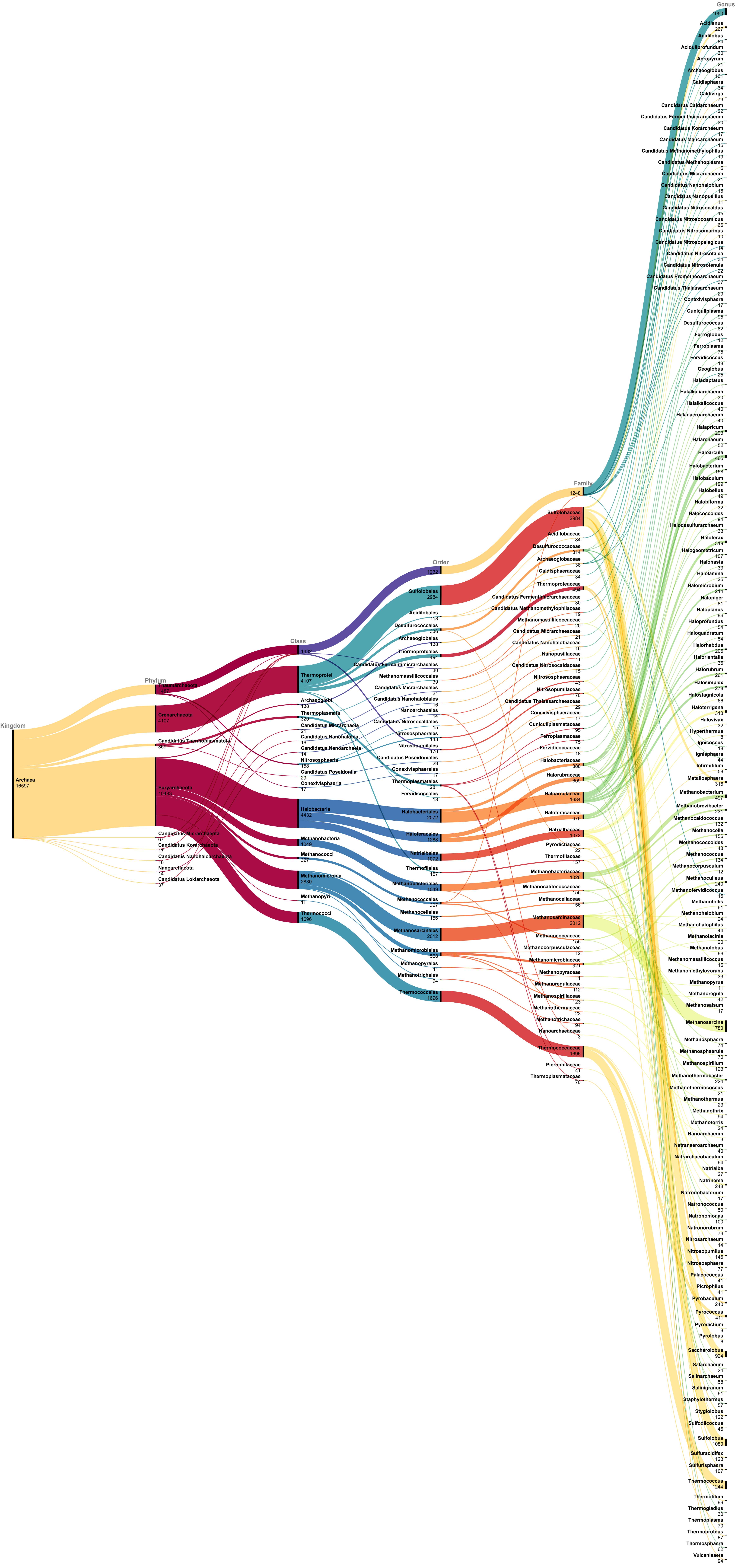

Kingdom
Archaea
16597
Phylum
Thaumarchaeota
1487
Crenarchaeota
4107
Candidatus Thermoplasmatota
369
Euryarchaeota
10483
Candidatus Micrarchaeota
67
Candidatus Korarchaeota
17
Candidatus Nanohaloarchaeota
16
Nanoarchaeota
14
Candidatus Lokiarchaeota
37
Class
1432
Thermoprotei
4107
Archaeoglobi
138
Thermoplasmata
320
Candidatus Micrarchaeia
21
Candidatus Nanohalobia
16
Candidatus Nanoarchaeia
14
Nitrososphaeria
158
Candidatus Poseidoniia
29
Conexivisphaeria
17
Halobacteria
4432
Methanobacteria
1049
Methanococci
327
Methanomicrobia
2830
Methanopyri
11
Thermococci
1696
Order
1232
Sulfolobales
2984
Acidilobales
118
Desulfurococcales
338
Archaeoglobales
138
Thermoproteales
494
Candidatus Fermentimicrarchaeales
30
Methanomassiliicoccales
39
Candidatus Micrarchaeales
21
Candidatus Nanohalobiales
16
Nanoarchaeales
14
Candidatus Nitrosocaldales
15
Nitrososphaerales
143
Nitrosopumilales
170
Candidatus Poseidoniales
29
Conexivisphaerales
17
Thermoplasmatales
281
Fervidicoccales
18
Halobacteriales
2072
Haloferacales
1288
Natrialbales
1072
Thermofilales
157
Methanobacteriales
1049
Methanococcales
327
Methanocellales
156
Methanosarcinales
2012
Methanomicrobiales
568
Methanopyrales
11
Methanotrichales
94
Thermococcales
1696
Family
1248
Sulfolobaceae
2984
Acidilobaceae
84
Desulfurococcaceae
314
Archaeoglobaceae
138
Caldisphaeraceae
34
Thermoproteaceae
494
Candidatus Fermentimicrarchaeaceae
30
Candidatus Methanomethylophilaceae
19
Methanomassiliicoccaceae
20
Candidatus Micrarchaeaceae
21
Candidatus Nanohalobiaceae
16
Nanopusillaceae
11
Candidatus Nitrosocaldaceae
15
Nitrososphaeraceae
143
Nitrosopumilaceae
170
Candidatus Thalassarchaeaceae
29
Conexivisphaeraceae
17
Cuniculiplasmataceae
95
Ferroplasmaceae
75
Fervidicoccaceae
18
Halobacteriaceae
388
Halorubraceae
609
Haloarculaceae
1684
Haloferacaceae
679
Natrialbaceae
1072
Pyrodictiaceae
22
Thermofilaceae
157
Methanobacteriaceae
1026
Methanocaldococcaceae
156
Methanocellaceae
156
Methanosarcinaceae
2012
Methanococcaceae
155
Methanocorpusculaceae
12
Methanomicrobiaceae
321
Methanopyraceae
11
Methanoregulaceae
112
Methanospirillaceae
123
Methanothermaceae
23
Methanotrichaceae
94
Nanoarchaeaceae
3
Thermococcaceae
1696
Picrophilaceae
41
Thermoplasmataceae
70
Genus
1050
Acidianus
267
Acidilobus
84
Aciduliprofundum
20
Aeropyrum
21
Archaeoglobus
101
Caldisphaera
34
Caldivirga
73
Candidatus Caldarchaeum
22
Candidatus Fermentimicrarchaeum
30
Candidatus Korarchaeum
17
Candidatus Mancarchaeum
16
Candidatus Methanomethylophilus
19
Candidatus Methanoplasma
5
Candidatus Micrarchaeum
21
Candidatus Nanohalobium
16
Candidatus Nanopusillus
11
Candidatus Nitrosocaldus
15
Candidatus Nitrosocosmicus
66
Candidatus Nitrosomarinus
10
Candidatus Nitrosopelagicus
14
Candidatus Nitrosotalea
34
Candidatus Nitrosotenuis
22
Candidatus Prometheoarchaeum
37
Candidatus Thalassarchaeum
29
Conexivisphaera
17
Cuniculiplasma
95
Desulfurococcus
82
Ferroglobus
12
Ferroplasma
75
Fervidicoccus
18
Geoglobus
25
Haladaptatus
1
Halalkaliarchaeum
30
Halalkalicoccus
40
Halanaeroarchaeum
40
Halapricum
293
Halarchaeum
52
Haloarcula
465
Halobacterium
158
Halobaculum
199
Halobellus
49
Halobiforma
32
Halococcoides
94
Halodesulfurarchaeum
33
Haloferax
319
Halogeometricum
107
Halohasta
33
Halolamina
25
Halomicrobium
214
Halopiger
81
Haloplanus
96
Haloprofundus
54
Haloquadratum
54
Halorhabdus
205
Halorientalis
35
Halorubrum
261
Halosimplex
278
Halostagnicola
66
Haloterrigena
318
Halovivax
32
Hyperthermus
8
Ignicoccus
18
Ignisphaera
44
Infirmifilum
58
Metallosphaera
316
Methanobacterium
497
Methanobrevibacter
231
Methanocaldococcus
132
Methanocella
156
Methanococcoides
48
Methanococcus
134
Methanocorpusculum
240
Methanoculleus
16
Methanofervidicoccus
16
Methanofollis
61
Methanohalobium
24
Methanohalophilus
44
Methanolacinia
20
Methanolobus
66
Methanomassiliicoccus
15
Methanomethylovorans
33
Methanopyrus
11
Methanoregula
42
Methanosalsum
17
Methanosarcina
1780
Methanosphaera
74
Methanosphaerula
70
Methanospirillum
123
Methanothermobacter
224
Methanothermococcus
21
Methanothermus
23
Methanothrix
94
Methanotorris
24
Nanoarchaeum
3
Natranaeroarchaeum
40
Natrarchaeobaculum
64
Natrialba
27
Natrinema
248
Natronobacterium
17
Natronococcus
50
Natronomonas
100
Natronorubrum
79
Nitrosarchaeum
14
Nitrosopumilus
146
Nitrososphaera
77
Palaeococcus
41
Picrophilus
41
Pyrobaculum
240
Pyrococcus
411
Pyrodictium
8
Pyrolobus
6
Saccharolobus
924
Salarchaeum
24
Salinarchaeum
58
Salinigranum
61
Staphylothermus
57
Stygiolobus
122
Sulfodiicoccus
45
Sulfolobus
1080
Sulfuracidifex
123
Sulfurisphaera
107
Thermococcus
1244
Thermofilum
99
Thermogladius
30
Thermoplasma
70
Thermoproteus
87
Thermosphaera
62
Vulcanisaeta
94

Supplementary Figure 3. Binding site structure comparison. Additional annotations of conserved active site and binding site residues in HX109_05010, TBR22_41900, HUW50_16055 and PDB 6GOC, 4CAG, 1DEO and 2W47.

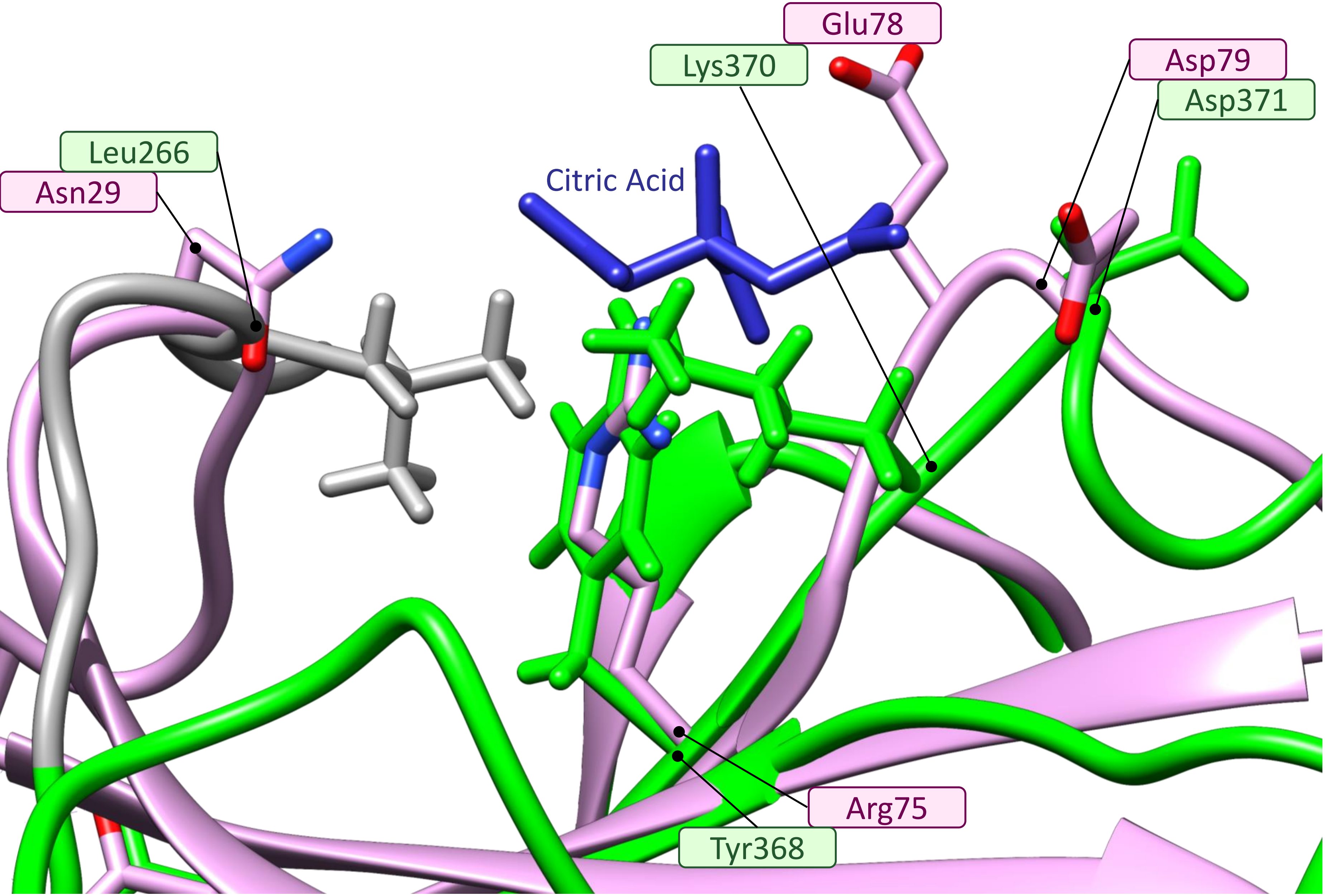

Supplementary figure 3a. Citric acid binding residues in PL20 enzyme PDB:2ZZJ and predicted structure fold of *HX109_05010*.

The structural fold of PDB:2ZZJ (shown in pink) superimposed onto the predicted structural fold (from Alphafold, v2.1.0) for the PL20 domain in HX109_05010 (shown in green, and disorganised region shown in grey). A citric acid in the PDB:2ZZJ structure is shown in blue. The side chains of residues in PDB:2ZZJ that bind citric acid and the corresponding residues in HX109_05010 are shown and annotated.

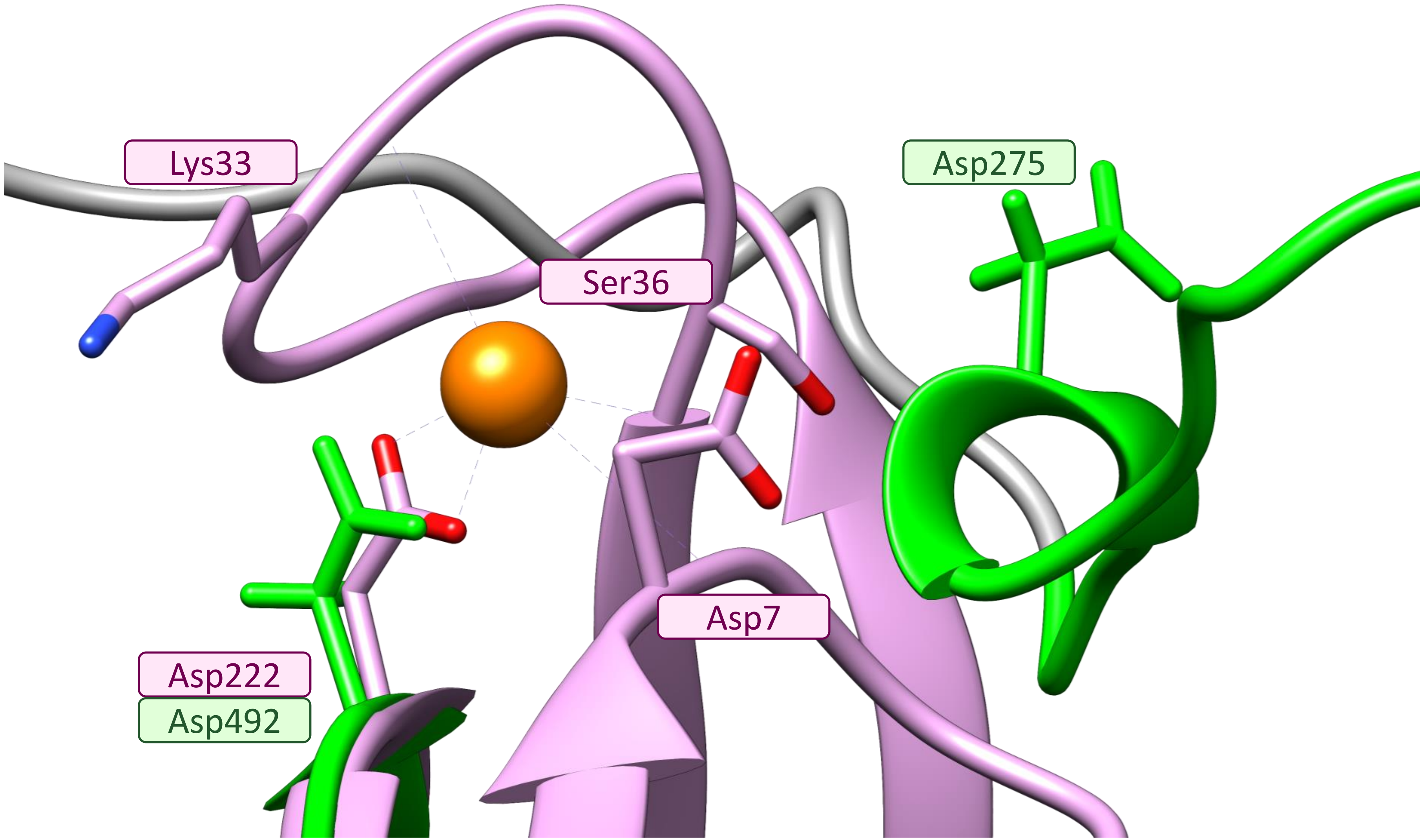

Supplementary figure 3b. Calcium ion binding residues in PL20 enzyme PDB:2ZZJ and predicted structure fold of *HX109_05010*.

The structural fold of PDB:2ZZJ (shown in pink) superimposed onto the predicted structural fold (from Alphafold, v2.1.0) for the PL20 domain in HX109_05010 (shown in green, and disorganised region shown in grey). A calcium ion in the PDB:2ZZJ structure is shown in orange. The side chains of residues in PDB:2ZZJ that bind calcium and the corresponding residues in HX109_05010 are shown and annotated.

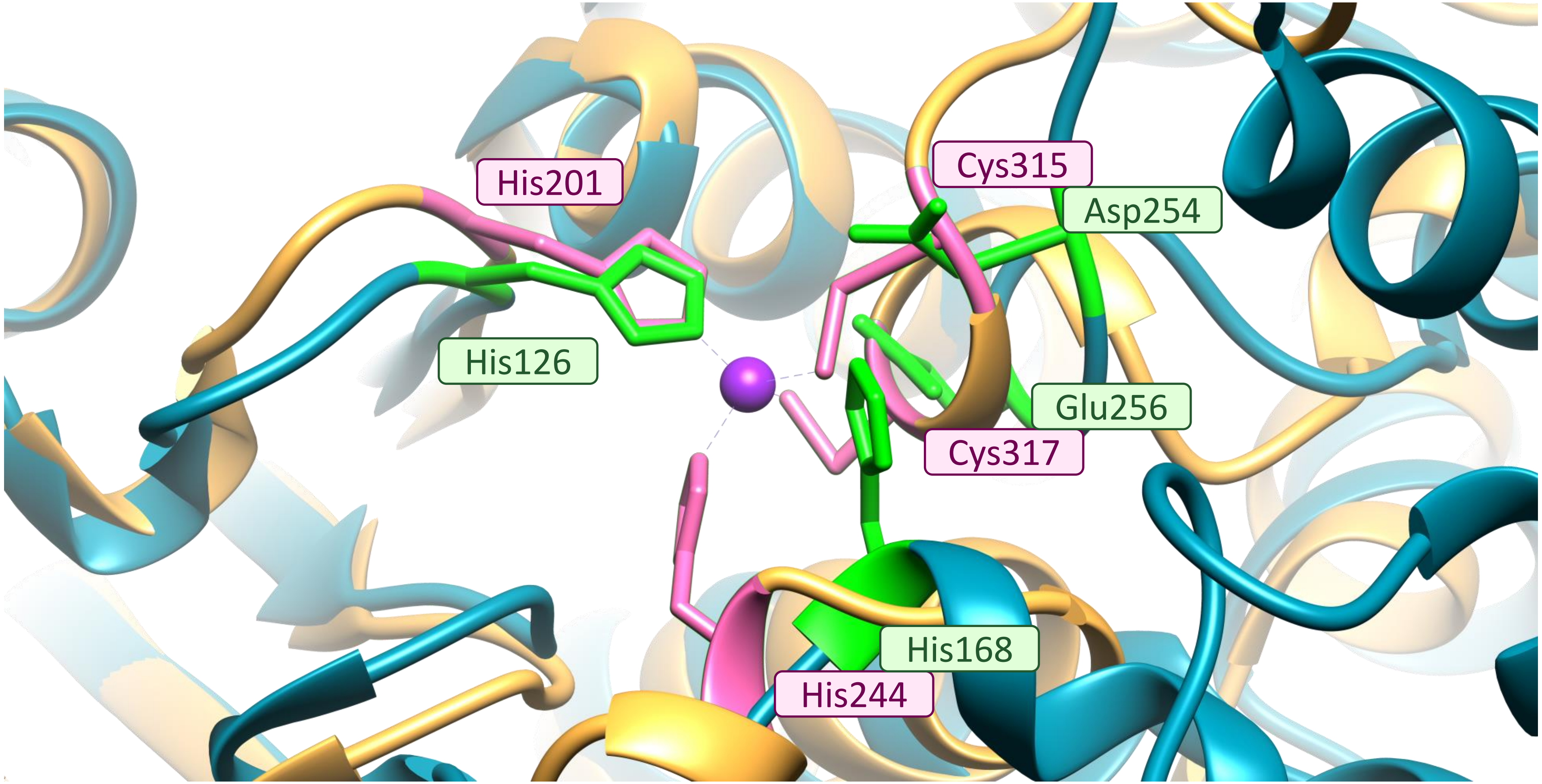

Supplementary figure 3c. Metal ion binding site in PDB:6GOC. *The metal ion (Zinc, shown in purple) binding site in PDB:6GOC (NCBI accession ALJ42174.1, shown in gold), superimposed onto the structural fold of TBR22_41900 (NCBI accession BCS34995.1) CE19 domain (predicted by alphafold), shown in blue, using Chimera MatchMaker tool. 6GOC metal binding residues are shown in pink; corresponding residues in TBR22_41900 are highlighted in green.*

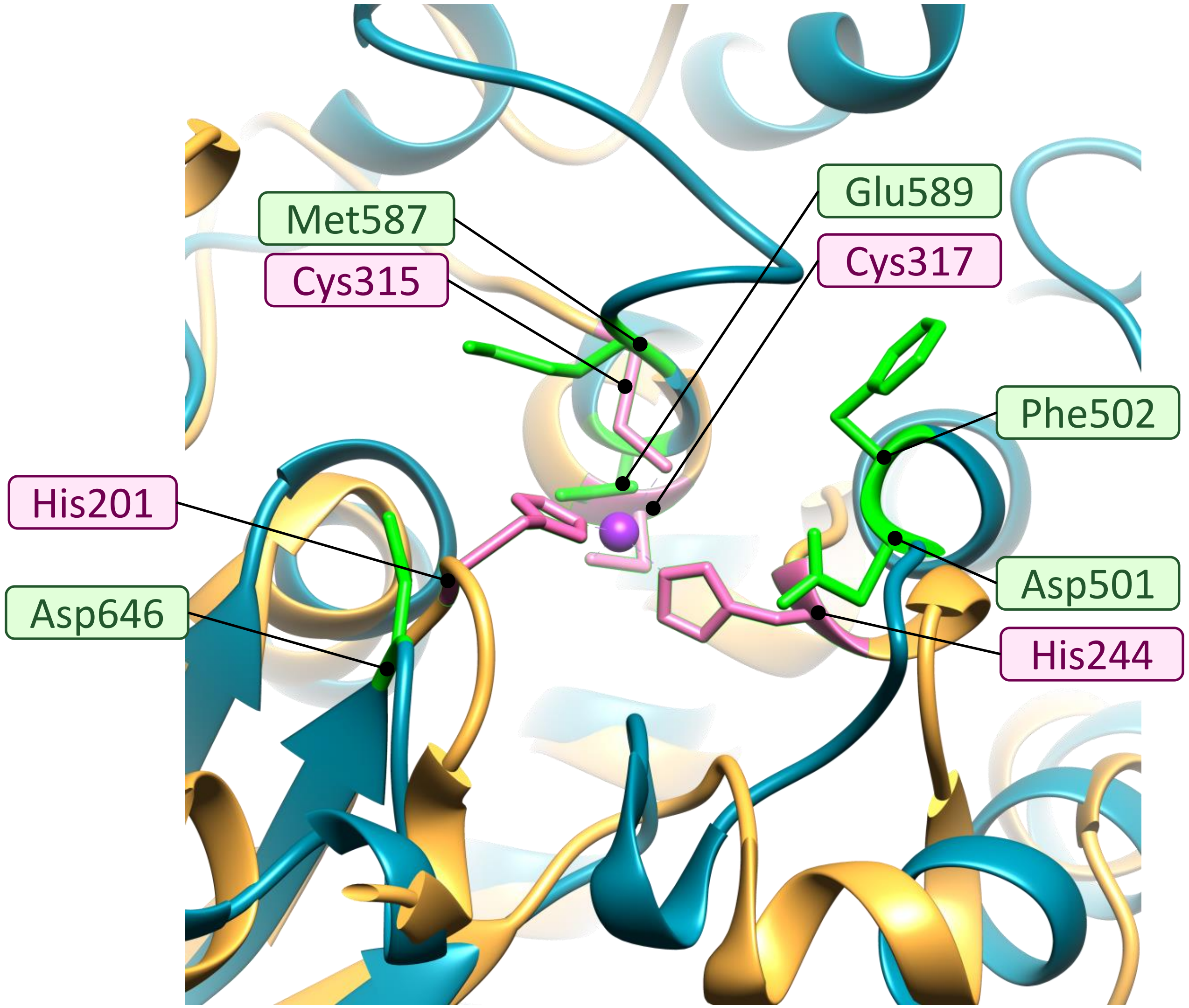

Supplementary figure 3d. Metal ion binding site in PDB:6GOC. The metal ion (Zinc, shown in purple) binding site in PDB:6GOC (NCBI accession ALJ42174.1, shown in gold), superimposed onto the structural fold of *TBR22_41900* (NCBI accession BCS34995.1) second domain (predicted by alphafold), shown in blue, using Chimera MatchMaker tool.. 6GOC metal binding residues are shown in pink; corresponding residues in *TBR22_41900* are highlighted in green.

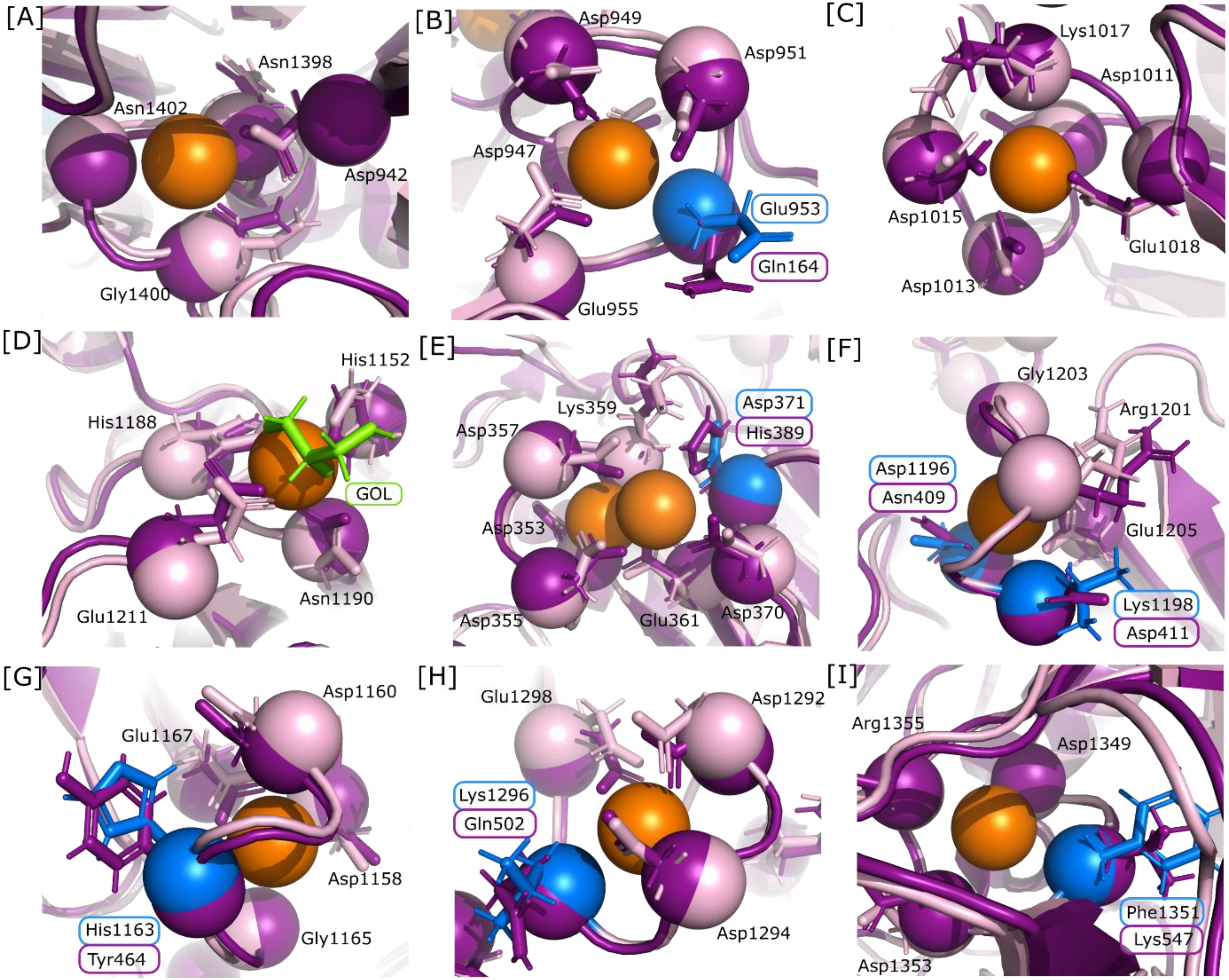

Supplementary figure 3e. Conserved calcium ion binding site residues in PL11 enzyme PDB:4CAG and predicted structural fold of *HUW50_16055*.

The structural fold of PDB:4CAG (shown in dark pink) superimposed onto the predicted structural fold (from Alphafold, v2.1.0) for the PL11 domain in HUW50_16055 (shown in light pink). Calcium ion binding residues in PDB:4CAG and corresponding residues in HUW50_16055 are shown as spheres with side chains shown as sticks. For residues that match, the position in HUW50_16055 is given. For residues that do not match, the residues in PDB:4CAG and HUW50_16055 are labelled, with the HUW50_16055 highlighted in blue.

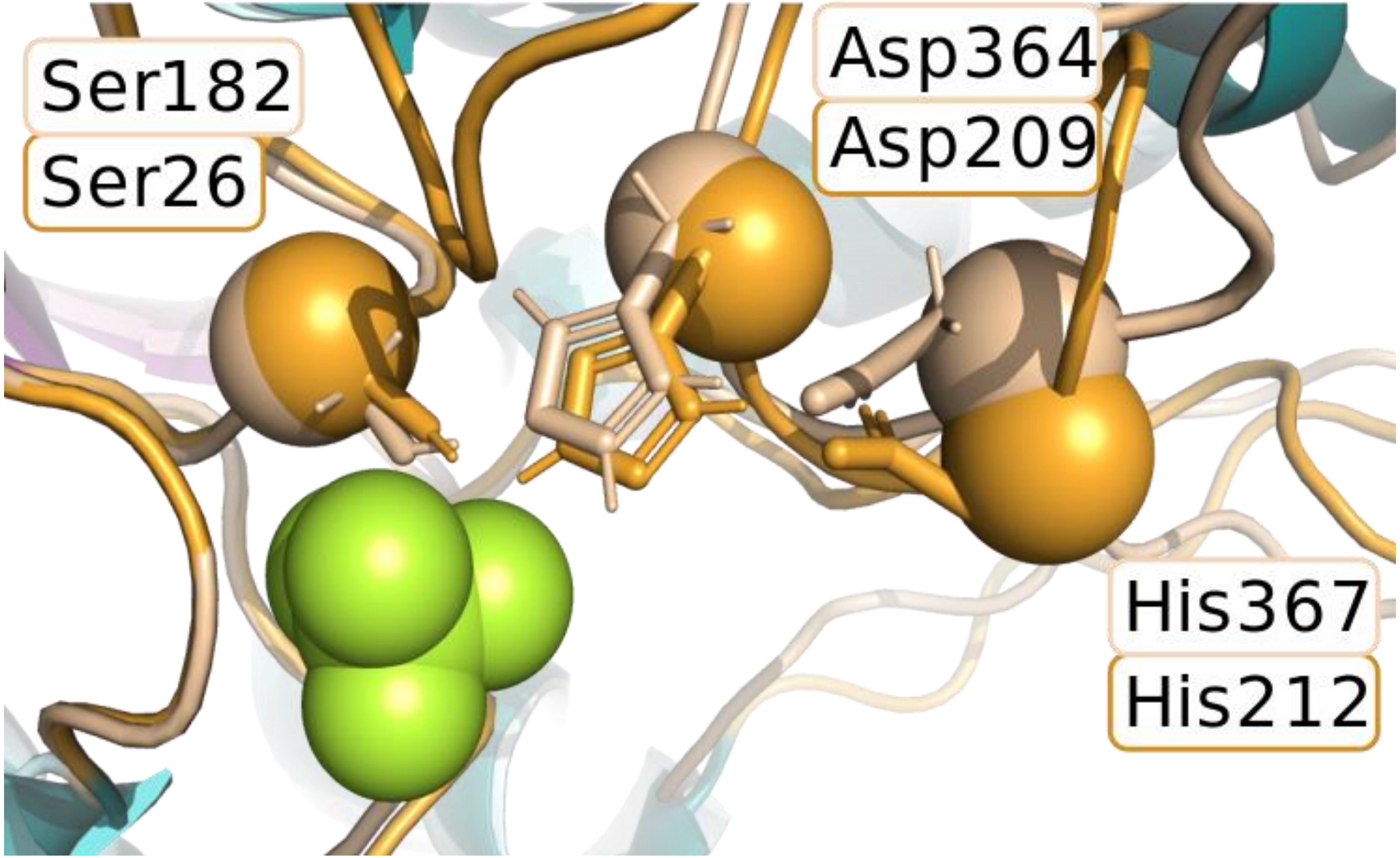

Supplementary figure 3f. Conserved binding site residues in CE12 enzyme PDB:1DEO and *HUW50_16055.*

PDB:1DEO (shown in darker shades) superimposed onto the predicted structural fold (from Alphafold, v2.1.0) for the CE12 domain in HUW50_16055 (shown in lighter shades). The catalytic triad in 1DEO and the corresponding residues in HUW50_16055 are shown as sphere with sidechains as sticks, and the residues are labelled.

Supplementary Figure 4. CE19 TBR22_41900 structure comparison. Result of superimposing PDB structures 6GOC, 3GY8, 3NUZ and 6RUI onto the predicted TBR22_41900 structure.

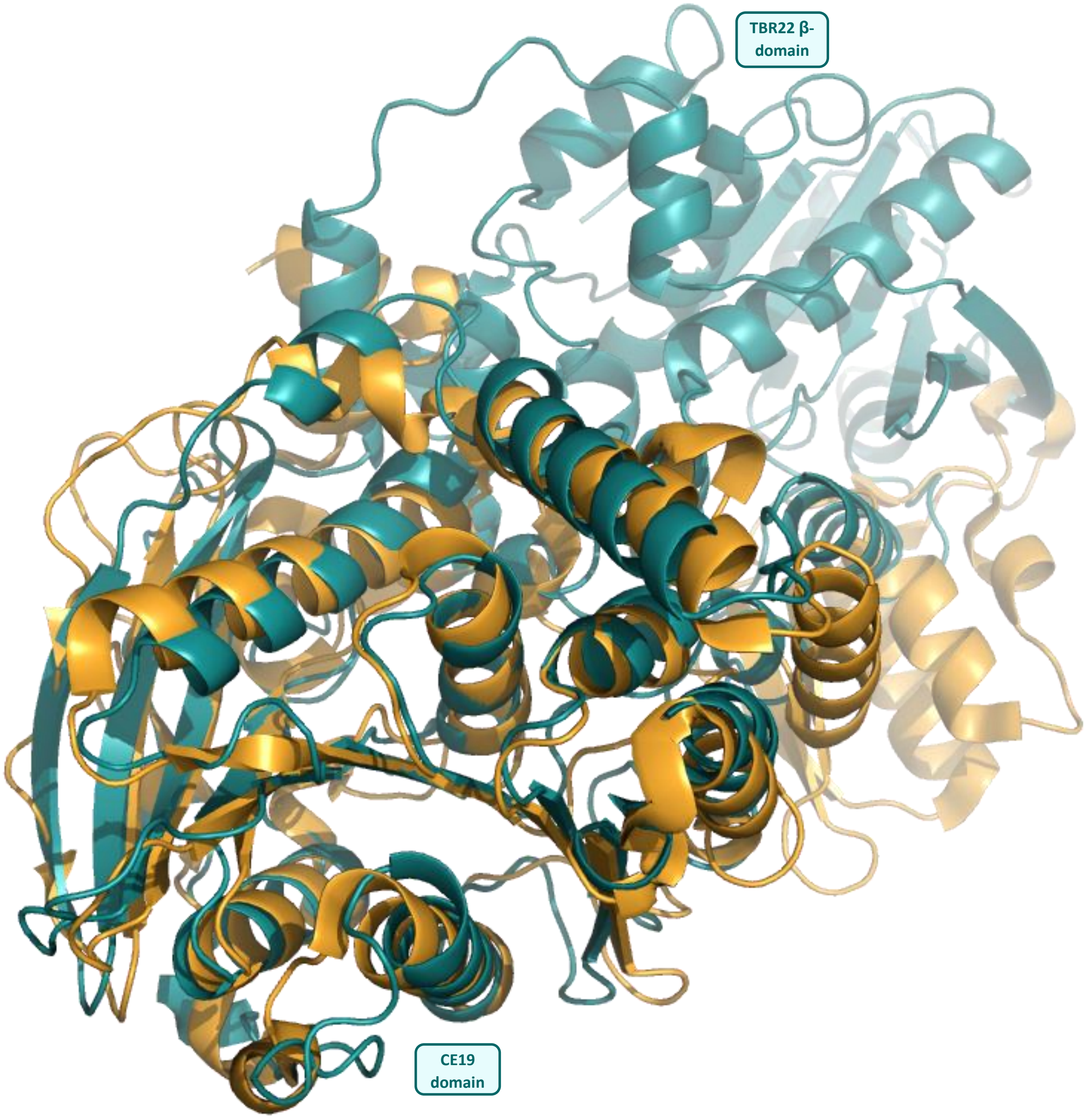

Supplementary Figure 4a. The PDB 6GOC structural fold (shown in orange) superimposed onto the structural fold of TBR22 predicted by alphafold (version 2.1.0) shown as secondary structures in teal. RMSD: 2.048Å (across 311 alpha carbons). 6GOC was aligned onto the TBR22 CE19 domain.

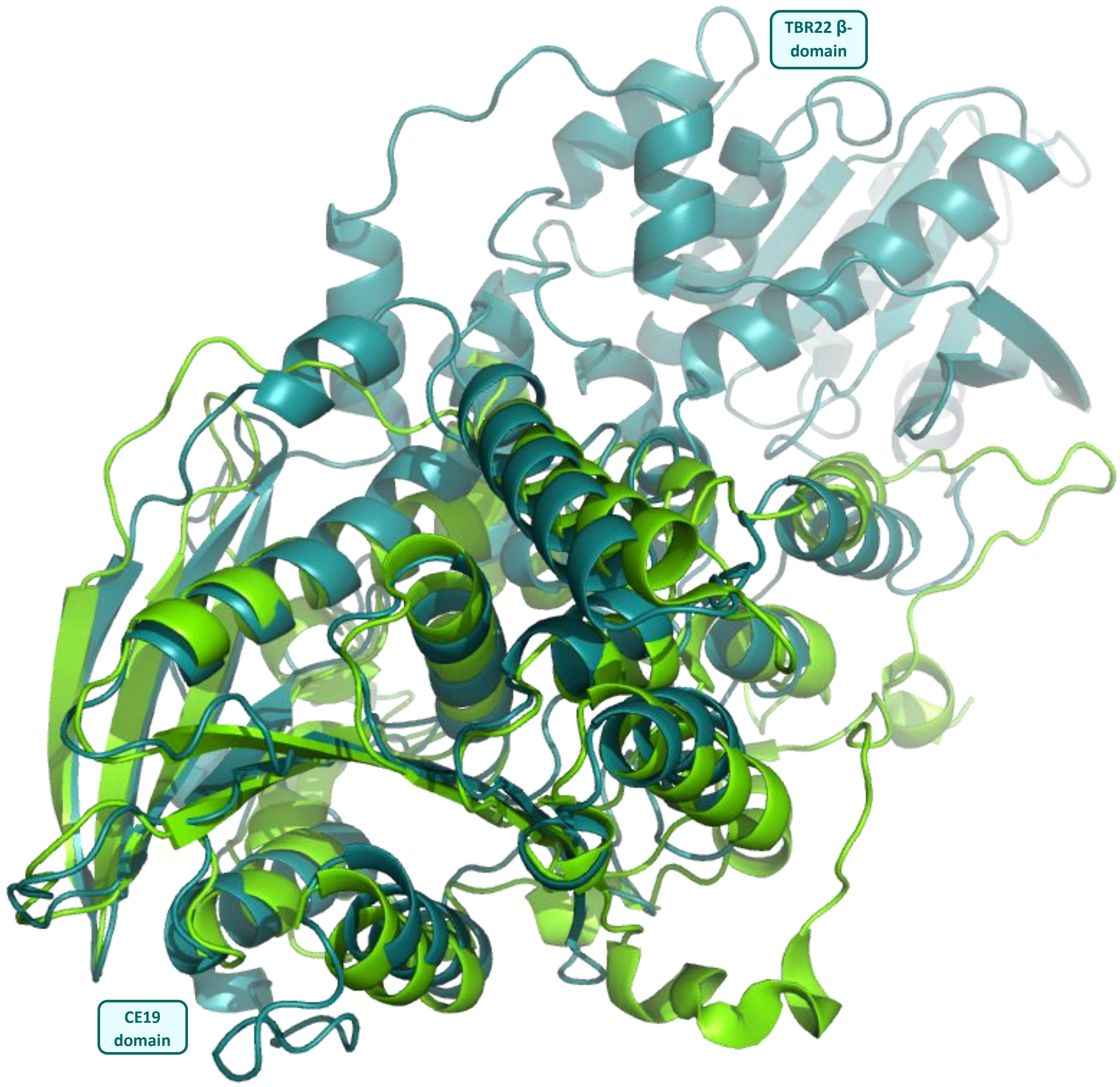

Supplementary Figure 4b. The PDB 3GY8 structural fold (shown in green) superimposed onto the structural fold of TBR22 predicted by alphafold (version 2.1.0) shown as secondary structures in teal. RMSD: 1.950 Å (across 278 alpha carbons). 3GY8 was aligned onto the TBR22 CE19 domain.

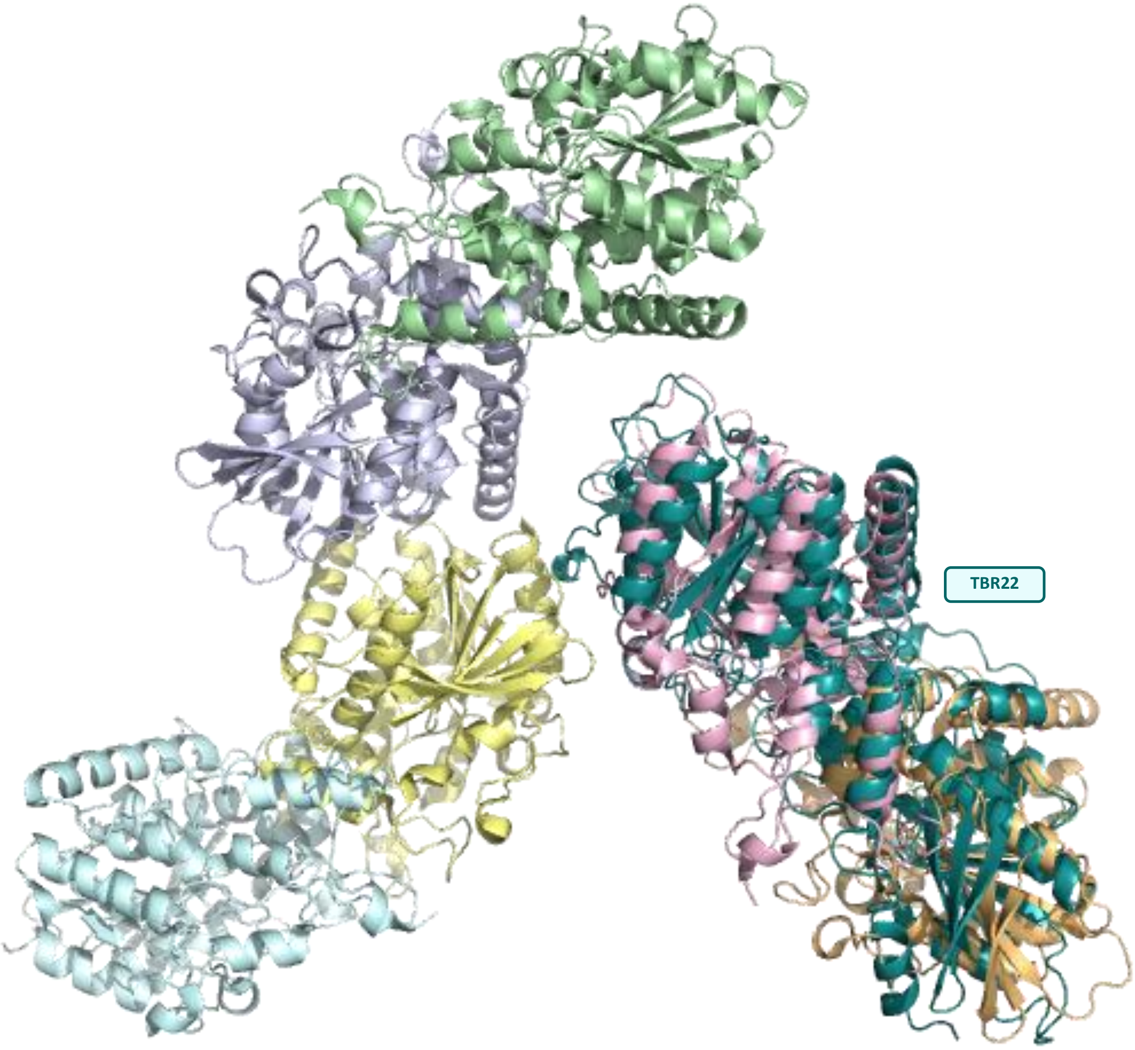

Supplementary Figure 4c. The PDB 3NUZ structural fold (chain A shown in green, chain B in clue, chain C in yellow, chain D in blue, chain E in pink and chain F in orange) superimposed onto the structural fold of TBR22 predicted by alphafold (version 2.1.0) shown as secondary structures in teal. RMSD: 1.852 Å (across 275 alpha carbons).

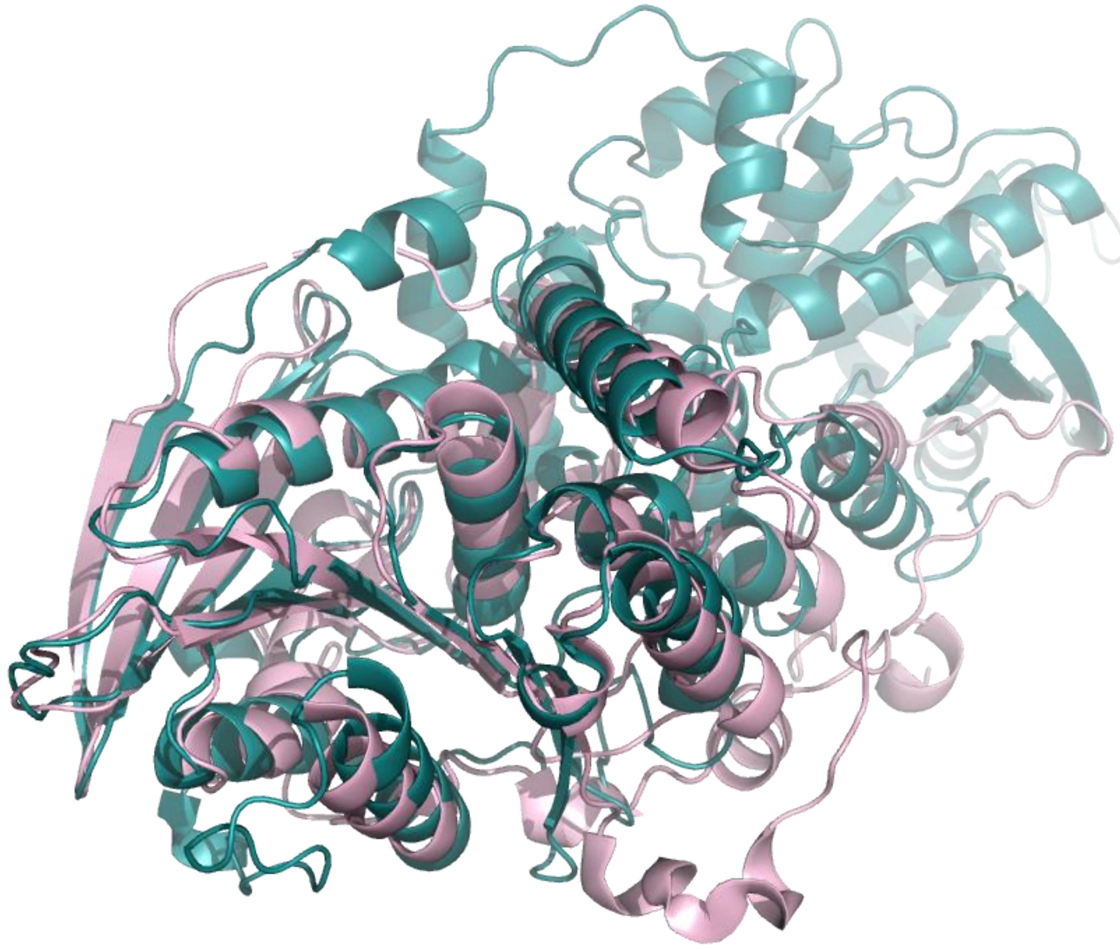

Supplementary Figure 4d. The PDB 3NUZ structural fold (chain E in pink) superimposed onto the structural fold of TBR22 predicted by alphafold (version 2.1.0) shown as secondary structures in teal. RMSD: 2.038 (863 to 863 atoms).

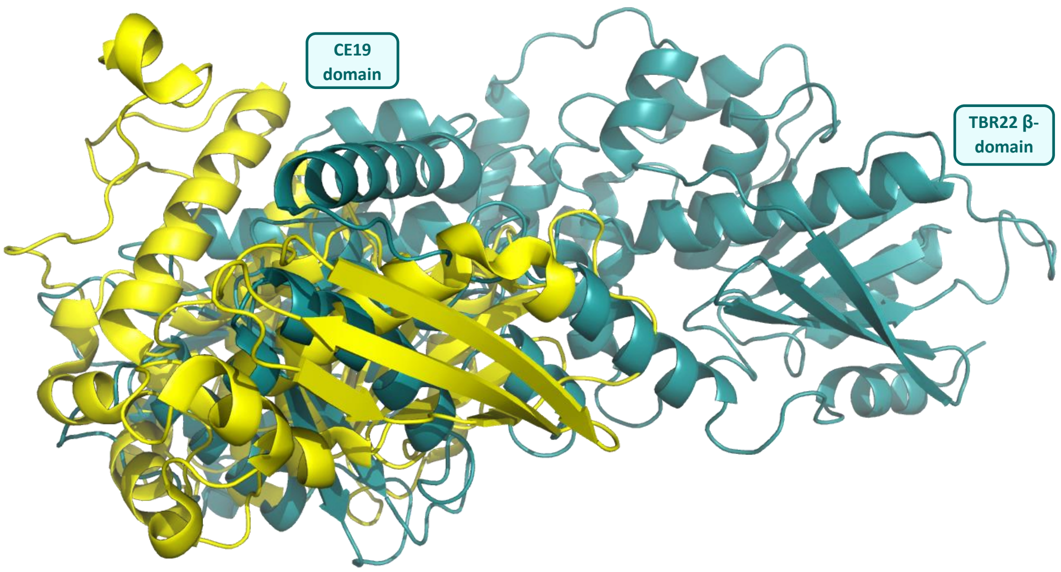

Supplementary Figure 4e. The PDB 6RUI structural fold (shown in yellow) superimposed onto the structural fold of TBR22 predicted by alphafold (version 2.1.0) shown as secondary structures in teal. RMSD: 2.234 Å (across 231 alpha carbons).

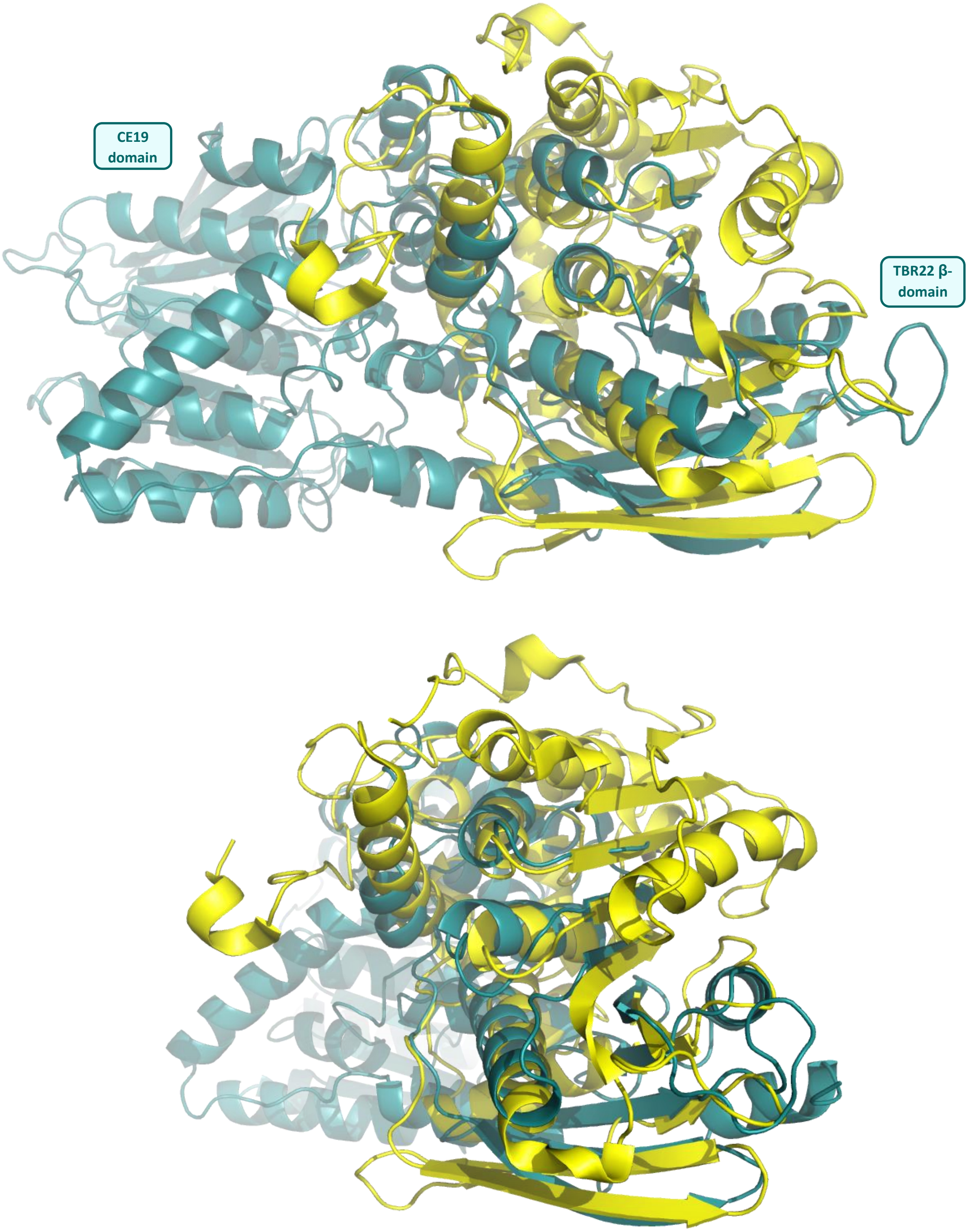

Supplementary Figure 4f. The PDB 6RUI structural fold (shown in yellow) superimposed onto the structural fold of TBR22 predicted by alphafold (version 2.1.0) shown as secondary structures in teal. RMSD: 2.352 Å (across 174 alpha carbons).

Table S1: *Comparison of operations (and functions) performed by tools that utilise data from the CAZy database.*

Operation performable by user	cazy_webscraper	CUPP	dbCAN	SACCHARIS	eggNOG
Compile local, queryable database	Y	-	-	-	-
Single step for installation (via package manager)	Y	-	-	-	n/a [1]
Retrieve protein annotation data from CAZy	Y	-[2]	-	-	Y
Annotate protein function	-	Y [3]	Y [3]	Y [4]	Y
Integrate genomic data from NCBI into the database	Y	-	-	-	-
Integrate taxonomic classifications from NCBI into the database	Y	Y	-	-	Y
Integrage taxonomic classifications from GTDB into the database	Y	-	-	-	-
Download structural data from PDB	Y	-	-	-	Y
Download and integrate UniProt protein IDs into the database	Y	-	-	-	-
Download and integrate EC numbers from UniProtKB into the database	Y	-	-	-	-
Predict EC numbers	-	Y	Y	-	-
Cluster protein sequences	-	Y [5]	-	Y	Y [6]
Construct gene trees		-	-	Y	Y

Available as a hosted database	-	Y[7]	- [8]	-	Y
Generates flat file of database	Y	Y	-	-	Y

[1] Not an installable tool

[2] The CUPP k-mer library (included in the tool) is pre-populated with CAZy family annotations

[3] Predicts CAZyme family annotations

[4] Uses dbCAN to predict CAZyme family annotations

[5] When compiling a new k-mer library

[6] Proteins provided already clustered into orthologous groups

[7] Pre-compiled k-mer library

[8] Can download HMM models and DIAMOND databases used by dbCAN
